# Publisher Correction: Environmental fluctuations accelerate molecular evolution of thermal tolerance in a marine diatom

**DOI:** 10.1038/s41467-018-05353-8

**Published:** 2018-07-13

**Authors:** C.-Elisa Schaum, A. Buckling, N. Smirnoff, D. J. Studholme, G. Yvon-Durocher

**Affiliations:** 1 Environment and Sustainability Institute, University of Exeter, Penryn Campus, Penryn, Cornwall, TR10 9EZ UK; 2 Institute for Hydrobiology and Fisheries, Section Oceanography, Hamburg University, 22767 Hamburg, Germany; 3 Biosciences, College of Life and Environmental Sciences, Geoffrey Pope Building University of Exeter, Exeter, EX4 4QD UK

Correction to: *Nature Communications*; 10.1038/s41467-018-03906-5, published online: 30 April 2018

The PDF version of this Article was updated shortly after publication following an error which resulted in the Φ symbol being omitted from the left hand side of equation 8. The HTML version was correct from the time of publication.

