# Supplementary material for: Environmental fluctuations accelerate molecular evolution of thermal tolerance in a marine diatom
Source: Nat Commun. 2018 Apr 30;9:1719. doi: 10.1038/s41467-018-03906-5 (PMC5928086; doi:10.1038/s41467-018-03906-5)
Supplement: Supplementary file 1 — Supplementary Information [file 41467_2018_3906_MOESM1_ESM.pdf]

Supplementary Information

For

Environmental fluctuations accelerate molecular evolution of thermal tolerance in a marine diatom

C.-Elisa Schaum, A. Buckling, N. Smirnoff, D. J. Studholme & G. Yvon-Durocher

Correspondence to elisa.schaum@uni-hamburg.de or g.yvon-durocher@exeter.ac.uk

This PDF file includes:

Supplementary Figures 1 – 15:

Supplementary Figure 1: Experimental Design
Supplementary Figure 2: Trajectories of population size throughout the selection experiment
Supplementary Figure 3: Light response curves for the photochemical efficiency of photosystem II.
Supplementary Figure 4: Changes in macromolecular composition per cell in ancestral and evolved samples
Supplementary Figure 5: Carbon-use efficiency in the lineages evolved under fluctuating warming.
Supplementary Figure 6: Neighbour joining tree based on Euclidean distance for single nucleotide variants in protein-coding regions that had reached fixation after 300 generations of evolution in the respective selection environment (see also main manuscript Fig. 4A)
Supplementary Figure 7: Single nucleotide variants in protein-coding regions that had reached fixation (upper row) and that had not yet reached fixation (lower row) after 300 generations of evolution in the respective selection evironment
Supplementary Figure 8: Neighbour joining tree based on Euclidean distance calculated from phenotypic trait values in the ancestor and evolved samples (see main manuscript Fig. 4b)
Supplementary Figure 9: Copy number variation (CNV) in chromosome 8 among populations
Supplementary Figure 10: CNV in chromosome 23 among populations

Supplementary Figure 11: CNV in chromosome 19 (THAPSchr_19a_19 genomic scaffold) among populations
Supplementary Figure 12: Estimated allele frequencies for 14 single-nucleotide variants that introduce premature stop codons into *T. pseudonana* protein-coding genes
Supplementary Figure 13: Estimated allele frequencies for single-nucleotide variants in seven *T. pseudonana* protein-coding genes that have recurrently undergone fixation of a non-silent single-nulceotide variant.
Supplementary Figure 14: Composition of bacterial communities across treatments (PCA of Bray-Curtis differences in bacterial composition across all treatments)
Supplementary Figure 15: Alternate version of Figure 1 (main manuscript) – per replicate trajectories of growth rate μ in the different treatments.

Supplementary Tables 1 - 17
Supplementary Table 1: Summary of traits in ancestral and evolved populations.
Supplementary Table 2: Model selection on generalised additive mixed effects model (GAMM) fitted to the trajectories of population growth.
Supplementary Table 3: Model selection on generalised additive mixed effects model (GAMM) fitted to the trajectories of population size.
Supplementary Table 4: Thermal tolerance curve parameters for the ancestor (growth rate).
Supplementary Table 5:. Model selection and parameters of thermal tolerance curves of the evolved lineages (growth rate).
Supplementary Table 6: Model selection and parameters for the thermal responses of gross photosynthesis and respiration in the ancestor.
Supplementary Table 7: Model selection and parameters for the thermal response of gross photosynthesis in the evolved lineages.
Supplementary Table 8: Model selection and parameters for the thermal response of respiration in the evolved lineages.
Supplementary Table 9: Model selection to determine the effects of selection regime on the carbon use efficiency.
Supplementary Table 10: Model selection for cell size, C, N, P, Si, RNA and protein content per biovolume, C:N, C:P, N:P, Chl:C ratio, well as ΦPSII at irradiance as in the incubator for the ancestor at assay temperatuers spanning 15ºC to 35ºC
Supplementary Table 11: Model selection for cell size, C, N, P, Si, RNA and protein content per biovolume, C:N, C:P, N:P, Chl:C ratio, well as ΦPSII at irradiance as in the incubator for evolved samples assayed in their selection environment.
Supplementary Table 12: PERMANOVA and pairwise comparison for treatment-level differences between treatments (based on SNVs)
Supplementary Table 13: PERMANOVA and pairwise comparison based on treatment-level divergence in phenotypic traits(Phenotypes)
Supplementary Table 14: Table of candidate genes in the populations from the fluctuating environment where variants re-occurred in independent replicate cultures subjected to elevated temperature nut not hose in those grown at moderate temperatures.
Supplementary Table 15: Aligned sequence depths for each sequenced population
Supplementary Table 16: Model selection for the light response curves of photochemical efficiency (FRRF data).

Supplementary Table 17: PERMANOVA and pairwise comparison for differences between treatments (Bacterial composition)

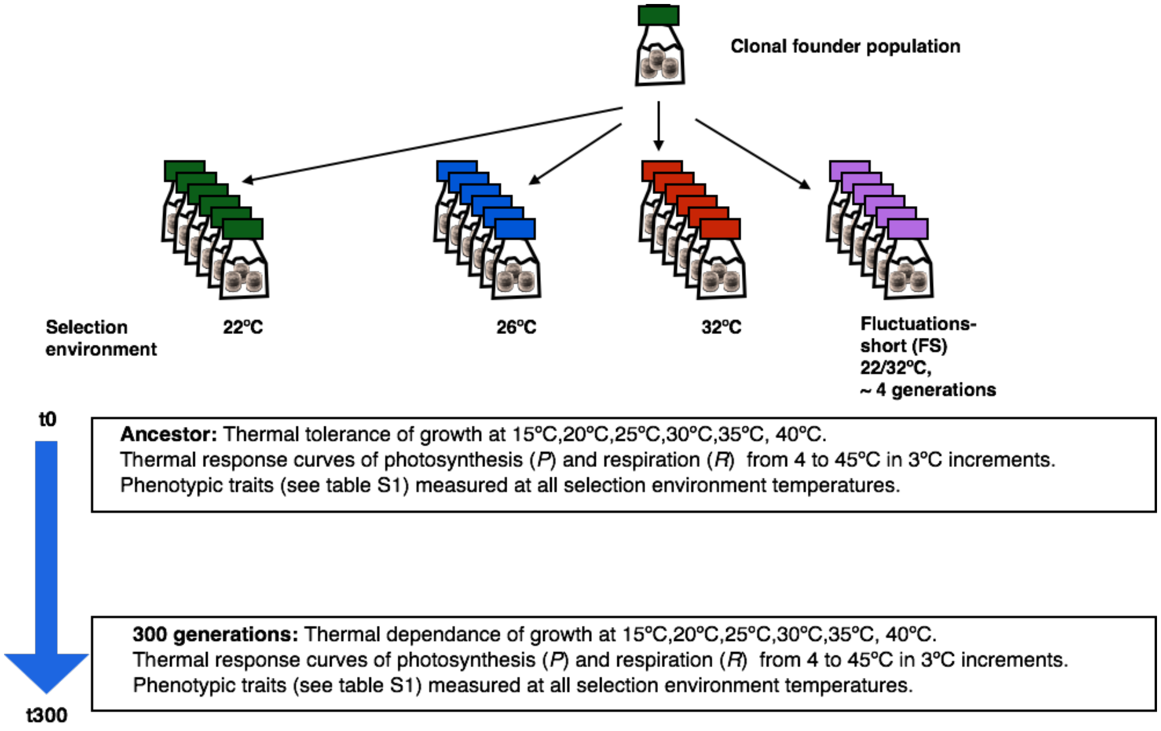

Supplementary Figure 1 | Experimental design. Six biological replicates in four selection regimes (the control environment at 22ºC, a moderate warming environment at 26ºC, a severe environment at 32ºC, and an environment that cycled between 22ºC and 32ºC approximately every 4 generations "fluctuating – short" or "FS") were founded from a single clone, and then propagated through semi-continuous batch culture for at least 300 generations. The temperatures for moderate and extreme warming were chosen based on pilot data, which showed that 32ºC was past the optimum temperature for growth, but did not induce excessive mortality, and that 26ºC represented the predicted average increase in sea surface temperature according to the IPCC RCP4.5 scenario (+ 4ºC from ambient). The fluctuating environment represents a conceptually more likely scenario where organisms' experience only short periods of severe conditions followed by recovery of the benign environment. At the beginning of the experiment (t0), and at the end (t300), a wide range of metabolic and macromolecular traits were quantified in the ancestor and the evolved lineages (see methods).

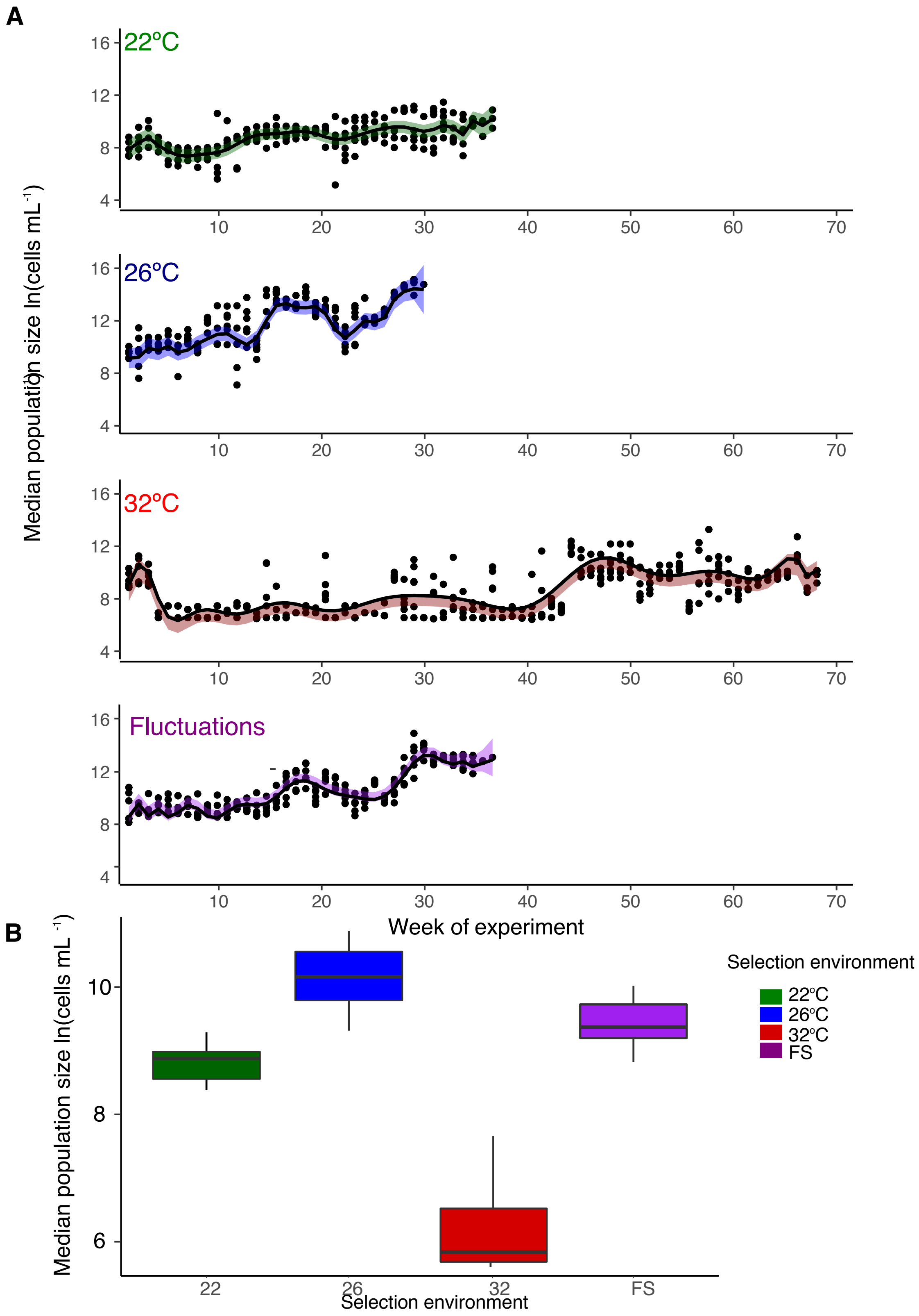

A
22ºC
26ºC
32ºC
Fluctuations
Median population size ln(cells mL-1)
Week of experiment
B
Median population size ln(cells mL-1)
Selection environment
Selection environment
22ºC
26ºC
32ºC
FS
22
26
32
FS

Supplementary Figure 2| Trajectories of population size throughout the selection experiment. (A) Trajectories of population size (displayed as natural logarithm of cells mL^{-1}) up to 300 generations determined from the cell density at the end of each transfer. Under moderate warming and in the fluctuating environment, there are rapid, sustained increases in population size. Under severe warming (32ºC), population size remained low until evolutionary rescue occurred after approximately 1 year (~ 100 generation). Although all samples received the same size inoculum at each transfer, mutational supply would have been larger in samples that attained higher population densities during the exponential phase of growth. Fitted lines are from the best fits of a GAMM (Supplementary Table 3). (B) Boxplots of replicate level estimates (fixed and random effects of GAMM) for median population size for each environment calculated over 300 generations. Median population size (again displayed as natural logarithms of cells mL^{-1}) was highest in samples evolving under moderate warming and in the fluctuating environment, while those at 32ºC had the lowest average population size. Green samples are the control at 22ºC, blue is 26ºC, red is 32ºC, and purple is the fluctuating environment. Boxplots are displayed so that whiskers indicate 1.5 interquartile range.

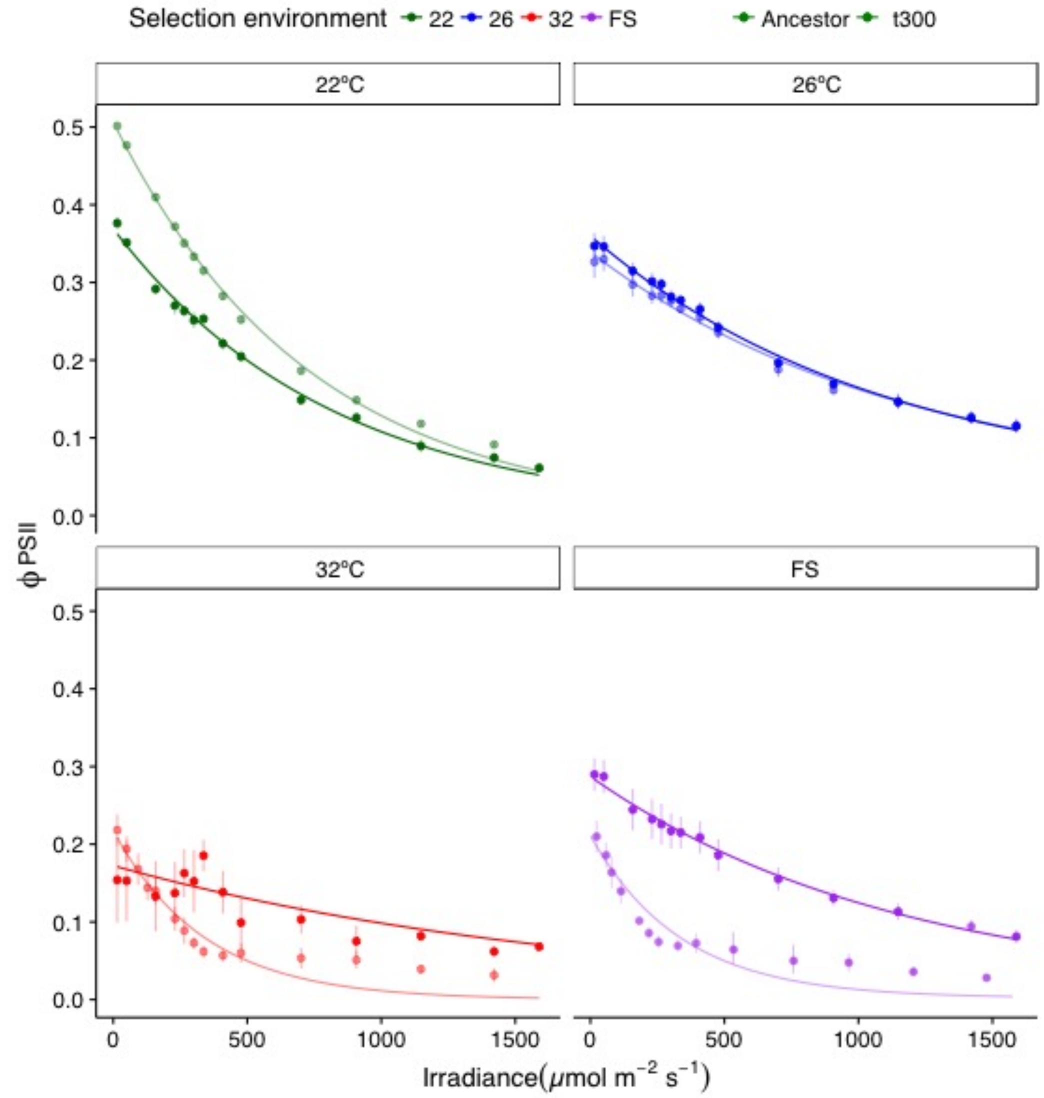

Supplementary Figure 3| Light response curves for the photochemical efficiency of photosystem II. The light response curves for photochemical efficiency, ΦPSII, differed both among selection environments and between the evolved lineages and the ancestor. ΦPSII was the highest and declined less steeply with increasing irradiance in the moderate (26ºC) and fluctuating warming treatments. Lineages in the severe warming treatment (32ºC) had very low photochemical efficiency. Green denotes populations evolved at 22ºC, blue for samples evolved at 26ºC, red for 32ºC, and purple for the fluctuating environment, FS. The ancestor (faded colour) at each temperature is displayed alongside the evolved lineages. All values are means ± 1s.e.m. The fitted curves are derived from the best fits of a non-linear mixed effects model on Eq. (8).

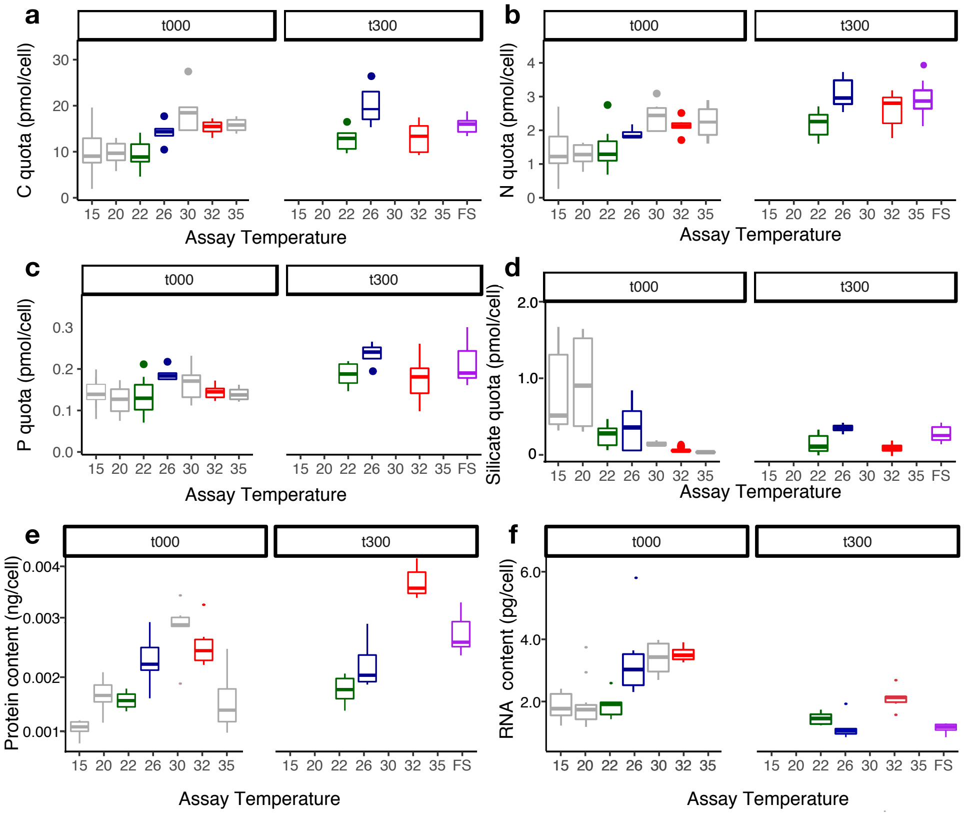

Supplementary Figure 4 | Changes in macromolecular composition per cell in ancestral and evolved samples. We investigated short-term thermal acclimation by exposing the ancestor at t000 to a 15 to 35ºC thermal gradient. The effects of long-term evolutionary adaptation to was quantified after 300 generations in the selection regimes**. (a-c)** For elemental stoichiometry, see main manuscript. The direction of acclimation was the same as that of the evolutionary response**, (d)** Intracellular silicate content decreased, on a per cell basis, with temperature in the short term, but samples at 26ºC and FS re-established silicate contents similar to those of the ancestor and the control after 300 generations. **(e-f):** Protein and RNA content per cell increased with temperature both in the short-term and in the long-term. For all boxplots, n=6. Grey denotes ancestor assay temperatures that were not used as selection regimes. Green for selection and/or assay at 22ºC, blue at 26ºC, red at 32ºC and purple for the fluctuating environment (FS). Boxplots are displayed so that whiskers indicate 1.5 interquartile range.

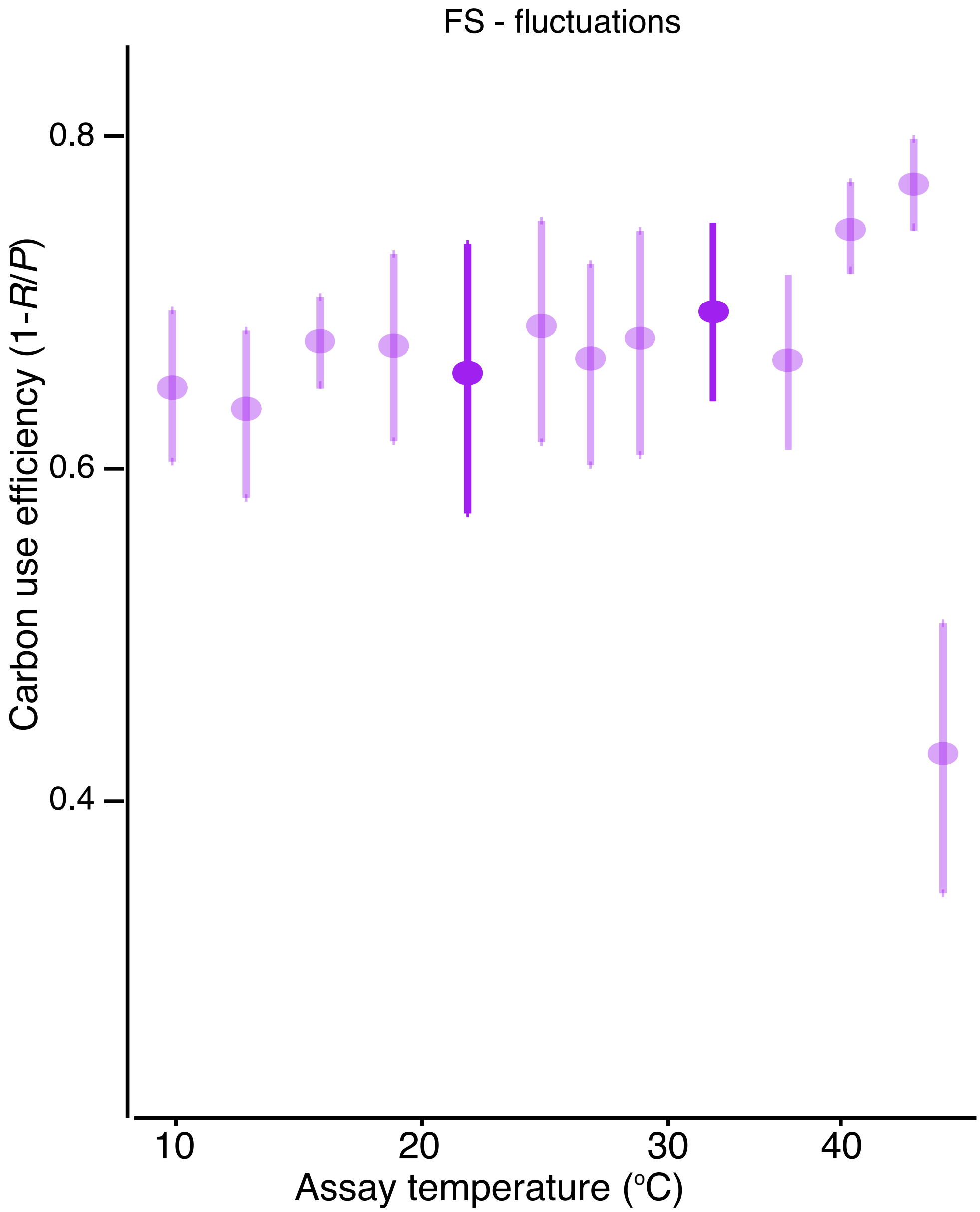

Supplementary Figure 5| Carbon-use efficiency in the lineages evolved under fluctuating warming. Carbon-use efficiency (CUE) in the lineages evolved in the fluctuating environment (between 22 and 32ºC) did not differ significantly between assay temperatures spanning 10ºC to 35ºC. In the main manuscript, we present CUE at 32ºC for ease of comparison with the stable 32ºC selection environment. 22ºC and 32ºC are in bold, all other assay temperatures are faded. Data are displayed as means ± 1 s.e.m.

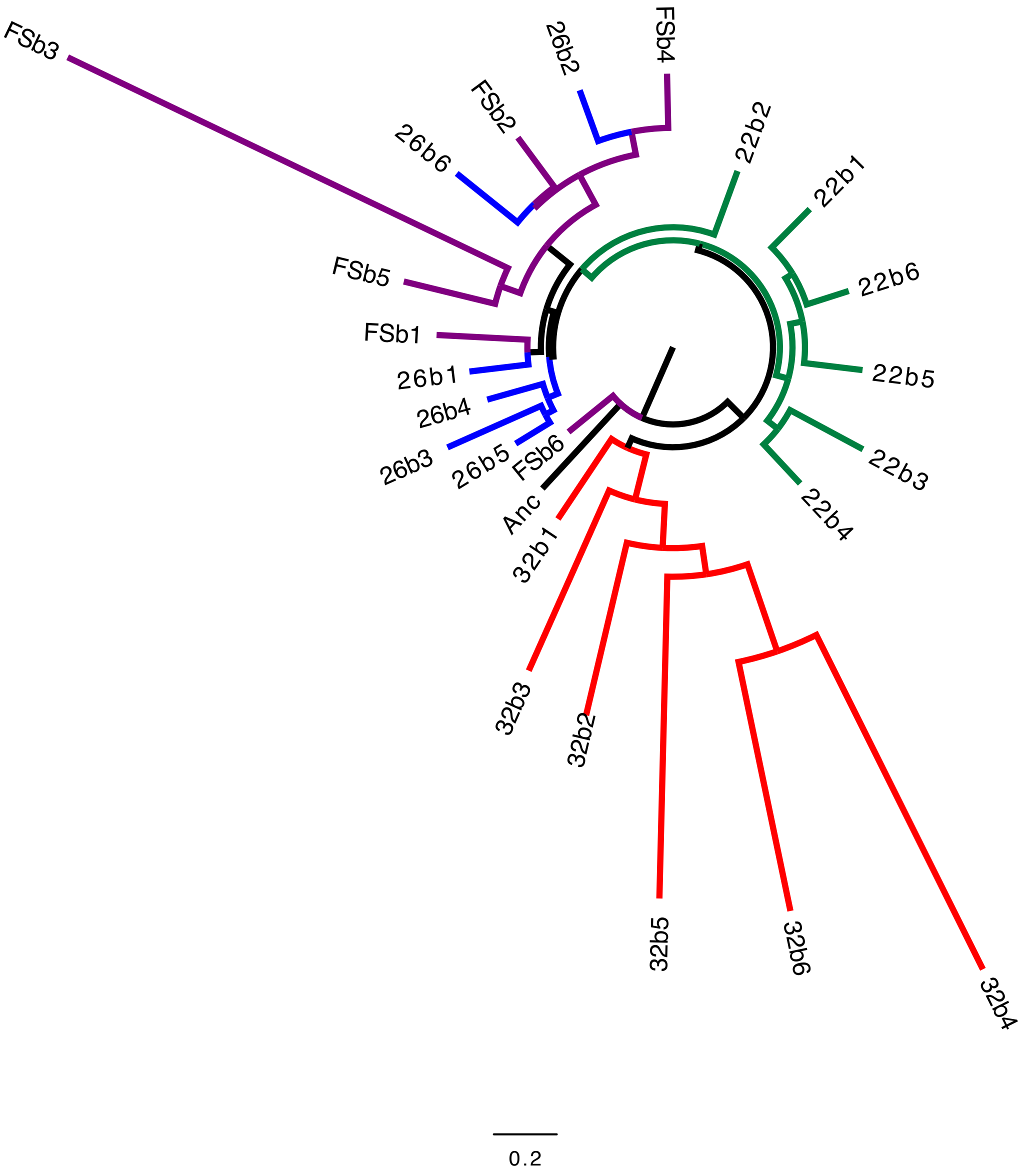

Supplementary Figure 6| Neighbour joining tree based on Euclidean distances for single nucleotide variants in protein-coding regions that had reached fixation after 300 generations of evolution in the respective selection environment. The tree has been rooted at the node including the ancestral population and shows clustering of samples evolved at 22ºC and 32ºC with samples from the 26ºC and fluctuating selection regime intertwined with each other. Evolved samples are colour coded based on selection regime with green denoting control (22ºC), blue, moderate warming (26ºC), red, severe warming (32ºC), and purple, evolution in the fluctuating environment. The bar is indicative of the Euclidean distance.

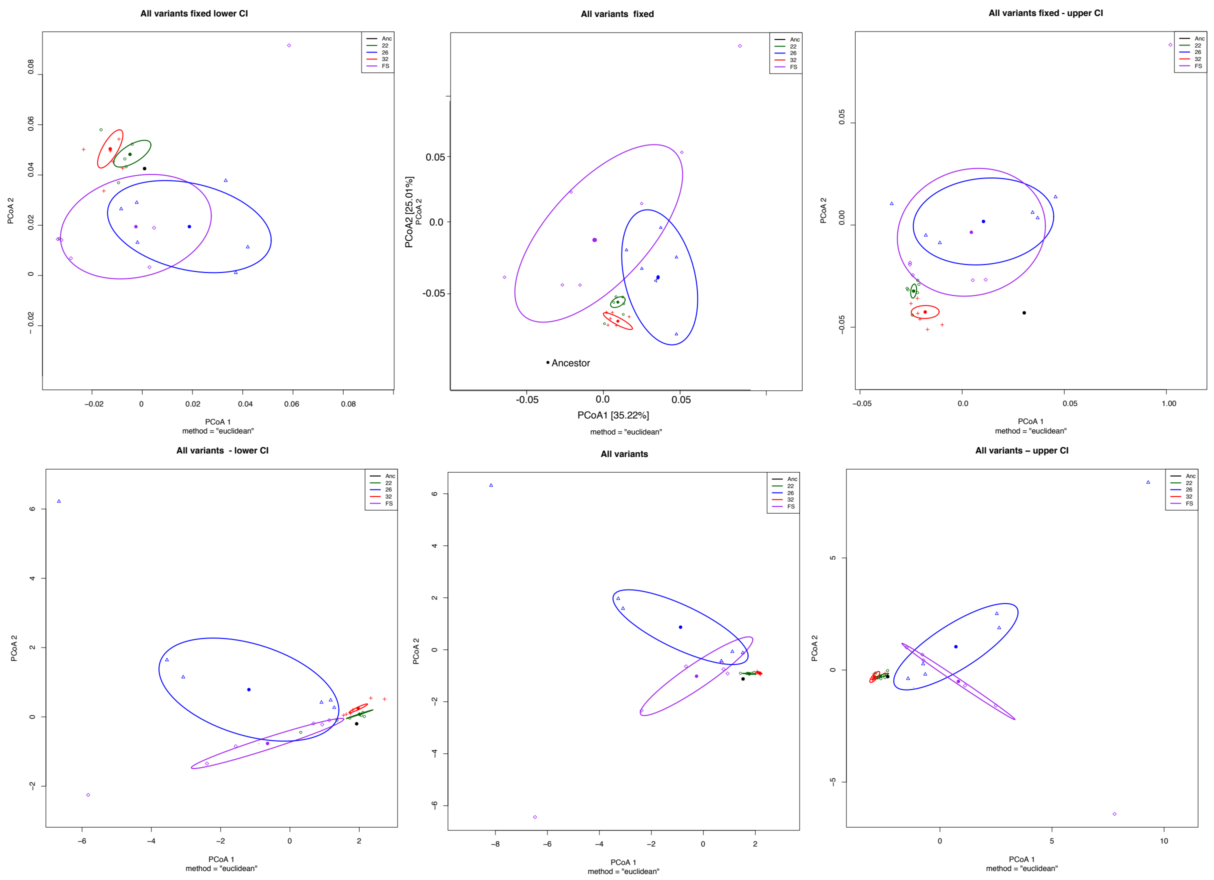

Supplementary Figure 7 | Single nucleotide variations in protein-coding regions that had reached fixation (upper row) and that had not yet reached fixation (lower row) after 300 generations of evolution in the respective selection environment. Displayed are the lower (left) and upper (right) confidence intervals around the estimate (middle), as calculated following Clopper & Pearson 1934 (see methods, reference 55). PCAs were constructed using Euclidean distance, and the colours denote the selection regime, with the Ancestor in black, populations evolved at 22ºC in green, blue for 26ºC, red for 32ºC, and purple for the fluctuating environment

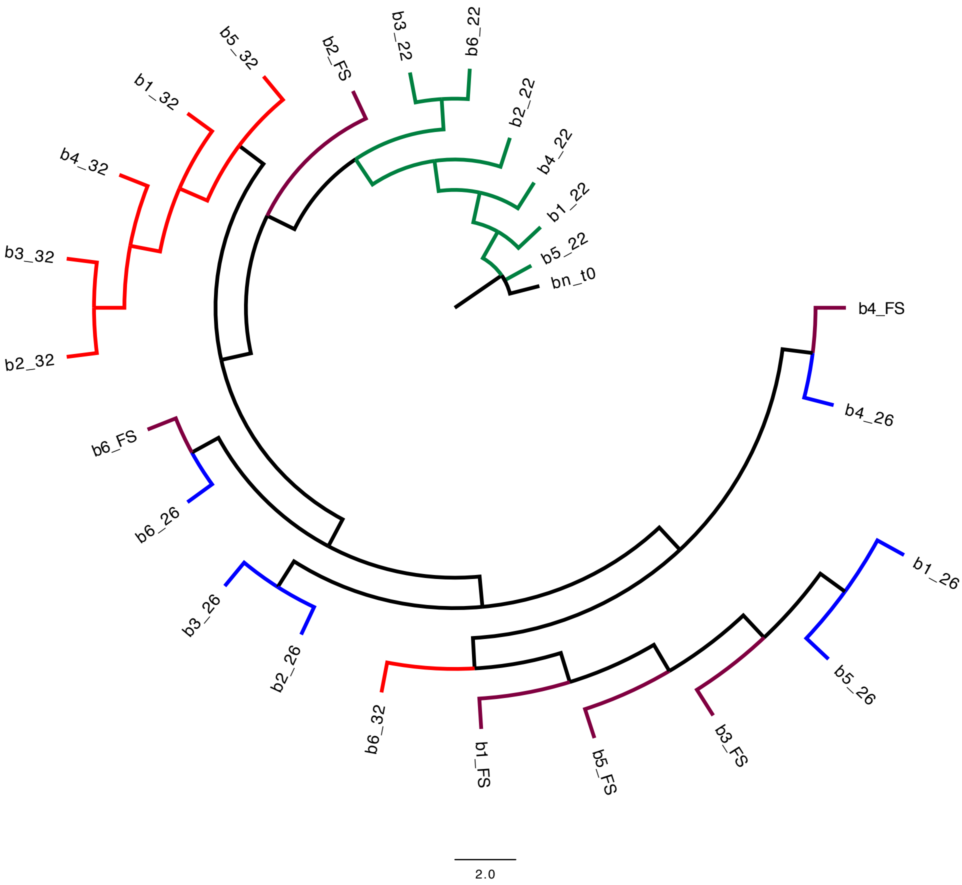

Supplementary Figure 8| Neighbour joining tree based on Euclidean distances calculated from phenotypic trait values in the ancestor and evolved samples. Samples evolved at 22ºC cluster with each other and are most similar to the ancestor, whereas samples from the 26ºC and fluctuating selection regime intertwined with each other and show a greater distance to the ancestor. Evolved samples are colour coded based on selection regime with green denoting control (22ºC), blue, moderate warming (26ºC), red, severe warming (32ºC), and purple, evolution in the fluctuating environment. The bar is indicative of the Euclidean distance.

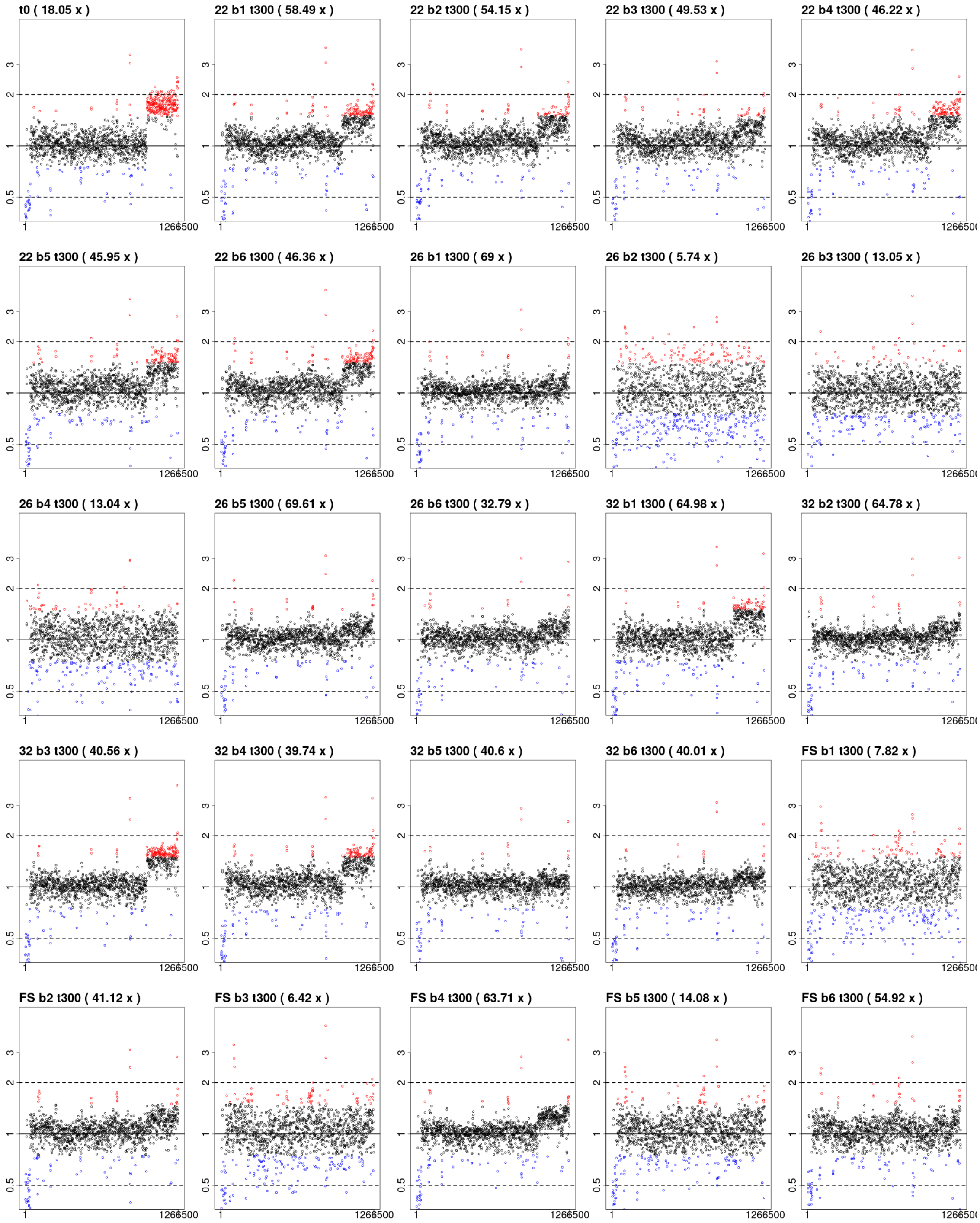

Supplementary Figure 9| Copy-number variation in chromosome 8 among populations. The horizontal axis represents position on the chromosome. The vertical axis represents sequencing depth normalized against the sequencing depth for that population over the whole genome. To aid visual identification of differences in copy-number profile among populations, depths of greater than 1.5 x median are colored red and those less than 0.75 x median are colored blue.

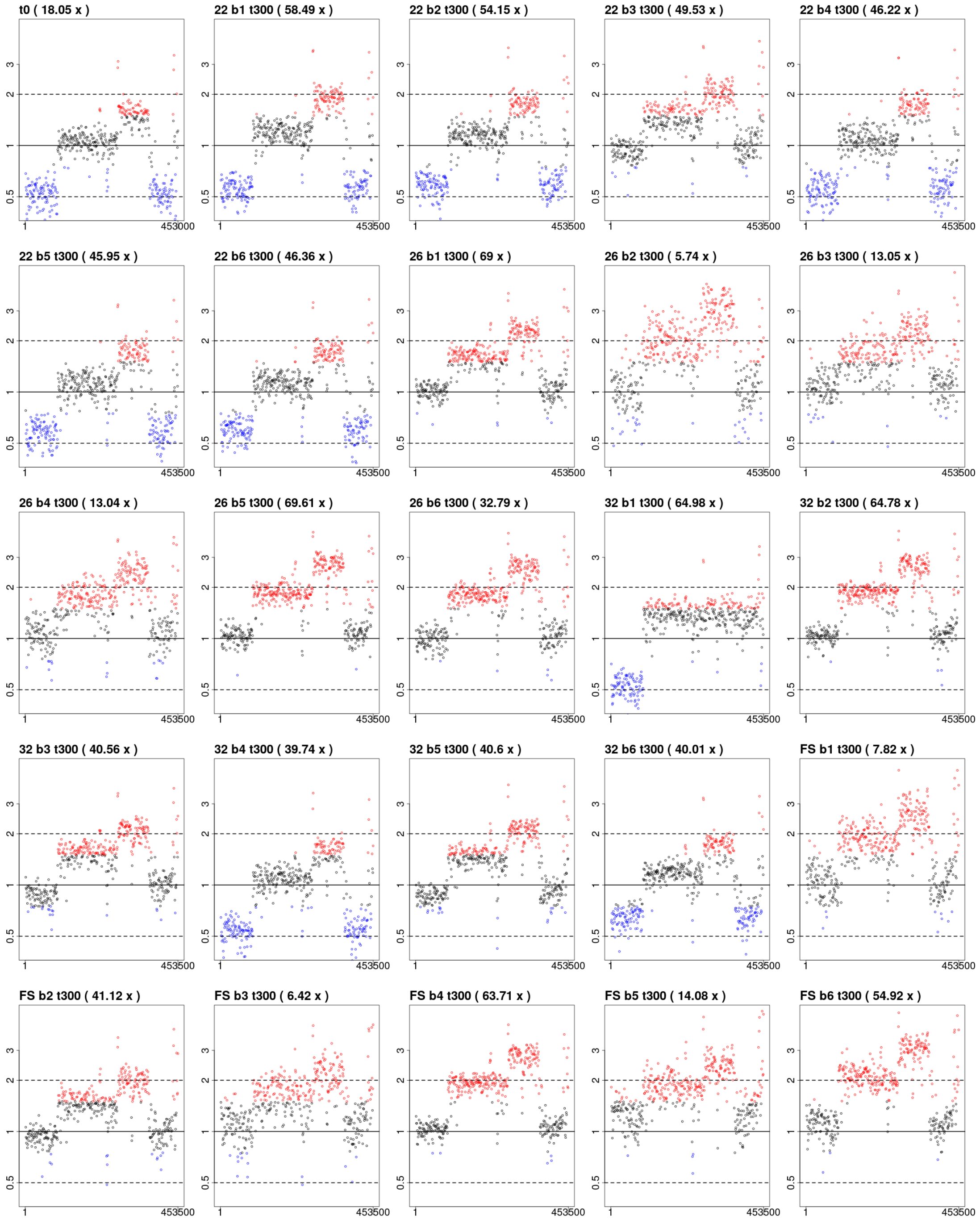

Supplementary Figure 10| Copy-number variation in chromosome 23 among populations. The horizontal axis represents position on the chromosome. The vertical axis represents sequencing depth normalized against the sequencing depth for that population over the whole genome. To aid visual identification of differences in copy-number profile among populations, depths of greater than 1.5 x median are colored red and those less than 0.75 x median are colored blue.

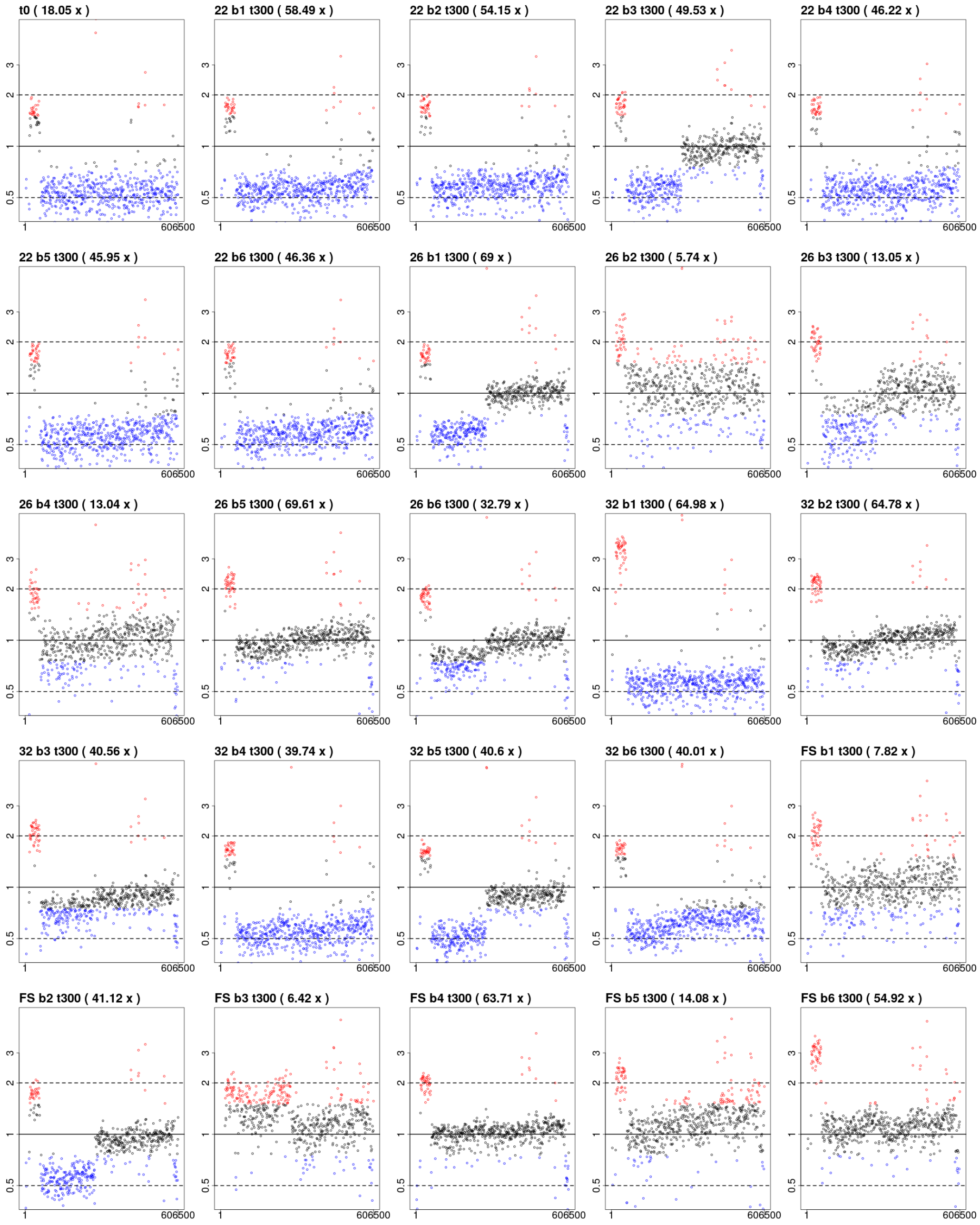

Supplementary Figure 11| Copy-number variation in chromosome 19 THAPSchr_19a_19 genomic scaffold among populations. The horizontal axis represents position on the chromosome. The vertical axis represents sequencing depth normalized against the sequencing depth for that population over the whole genome. To aid visual identification of differences in copy-number profile among populations, depths of greater than 1.5 x median are colored red and those less than 0.75 x median are colored blue.

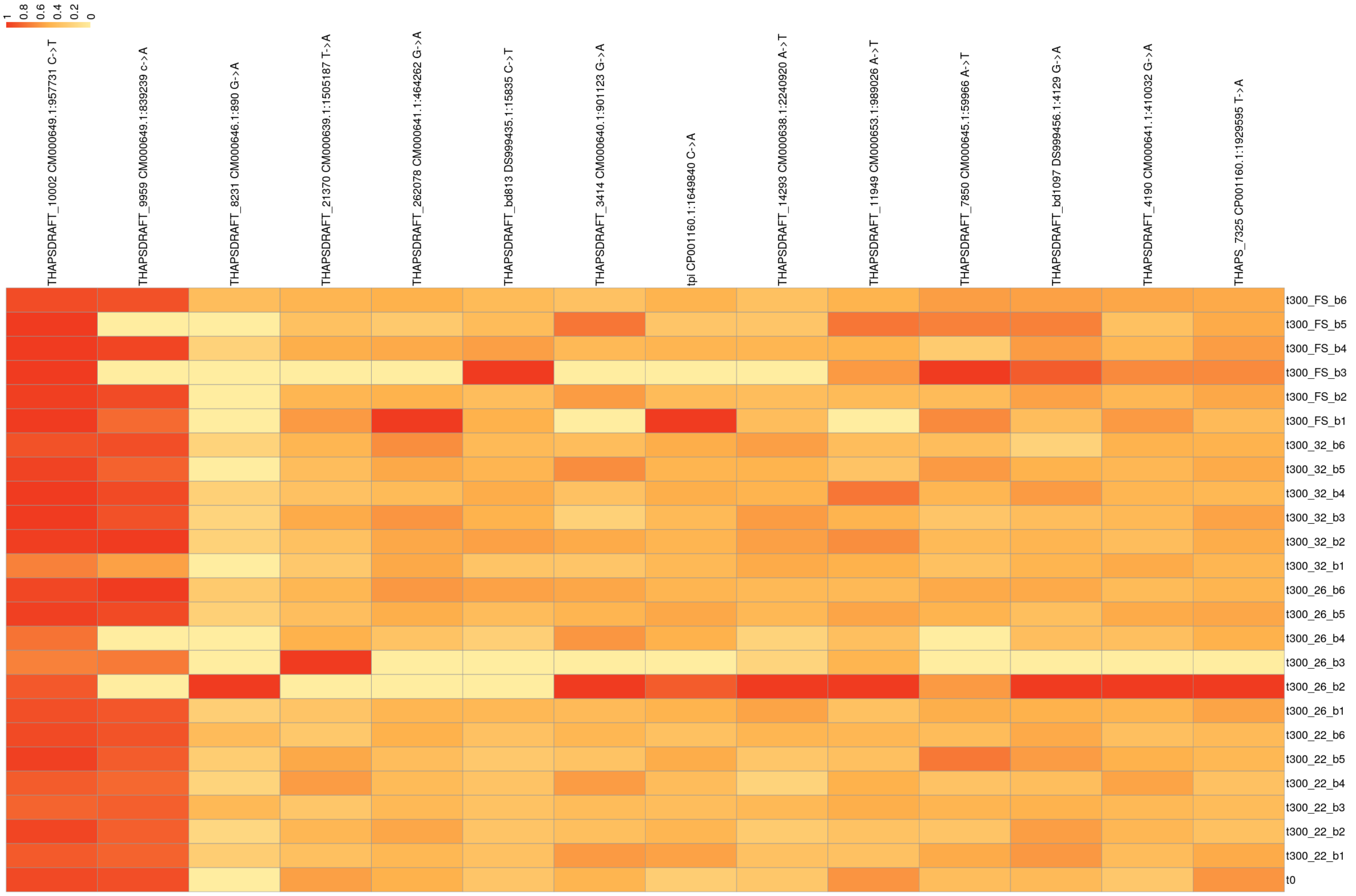

Supplementary Figure 12| Estimated allele frequencies for 14 single-nucleotide variants that introduce premature stop codons into *T. pseudonana* protein-coding genes. Each variant had putatively reached fixation in at least one t300 population; i.e. the estimated allele proportion was 1 in at least one of these populations. Colour of each cell in the heat map indicates estimated allele proportion in the population, based on ratio of variant sequence reads versus total read depth at that genomic site. The colour range is such that homozygously fixed alleles appear as yellow or red while heterozygously fixed alleles will appear as orange.

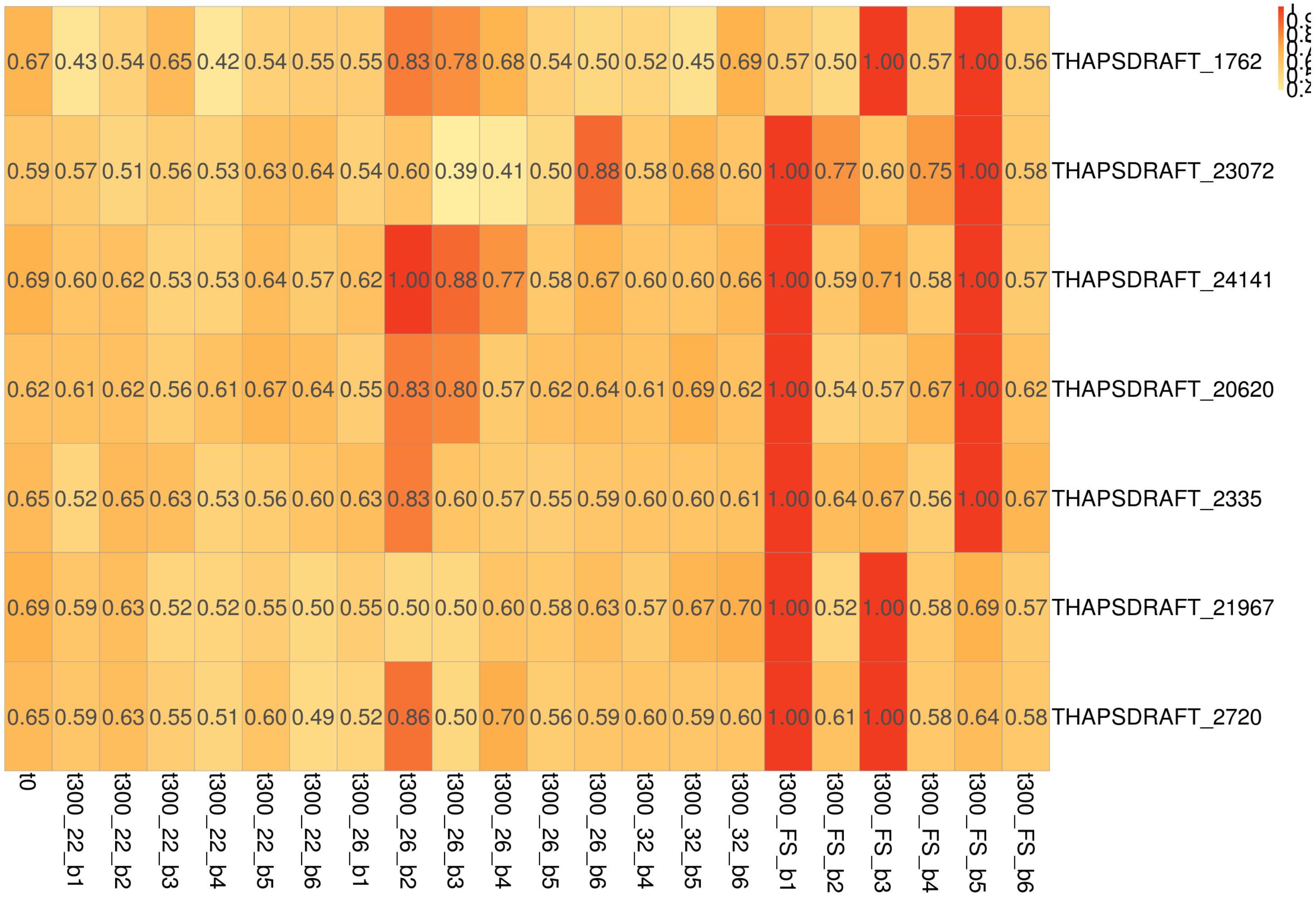

Supplementary Figure 13| Estimated allele frequencies for single-nucleotide variants in seven *T. pseudonana* protein-coding genes that have recurrently undergone fixation of a non-silent single-nucleotide variant. Each variant had putatively reached fixation in more than one t300t (32°C or FS) population; that is the estimated allele proportion was 1 in two of these populations. Color of each cell in the heat map indicates estimated allele proportion in the population, based on ratio of variant sequence reads versus total read depth at that genomic site. The color range is such that homozygously fixed alleles will appear as yellow or red while heterozygously fixed alleles appear as orange. Variants have been aggregated by gene, such that each column represents one gene and where more than one variant occurs in a single gene in a single population, the color indicates the proportion of the variant that is most abundant in that population.

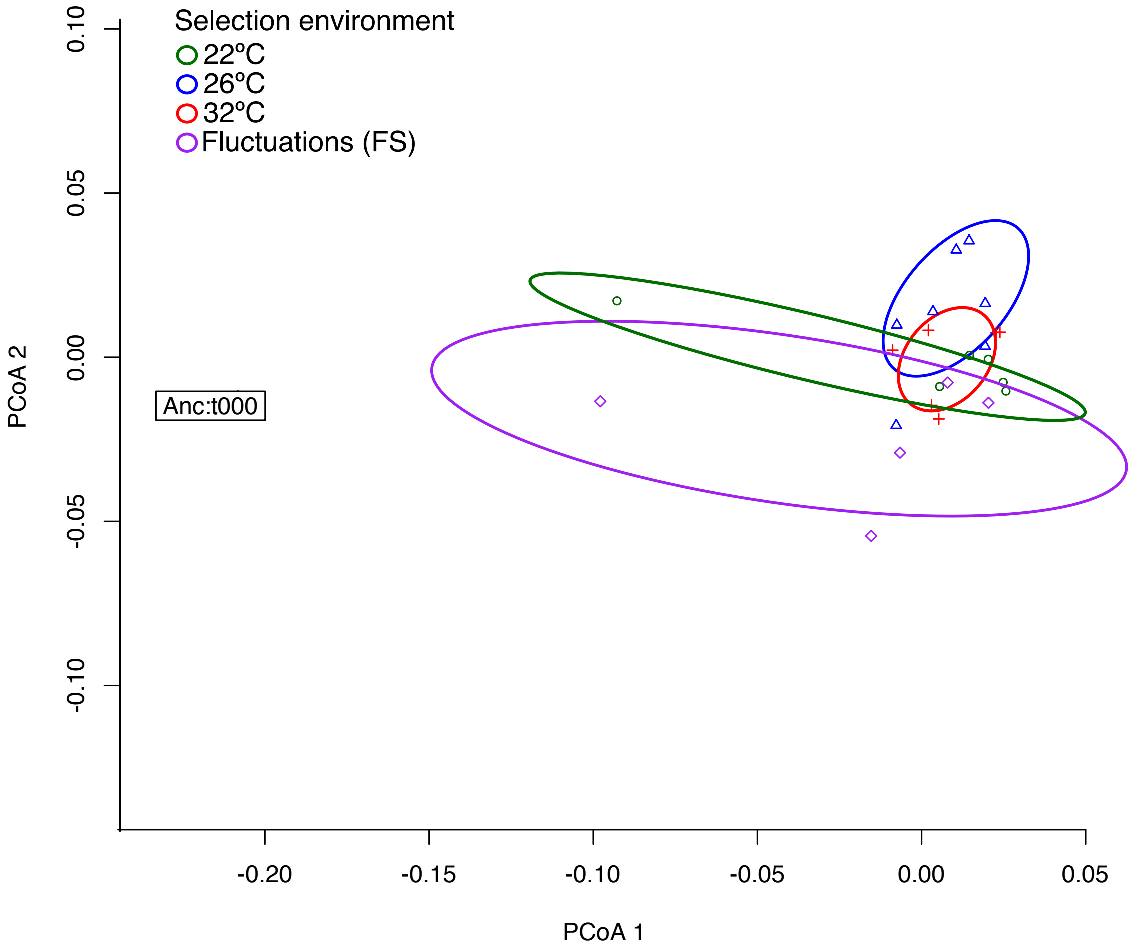

Supplementary Figure 14| Composition of bacterial communities across treatments. Principal components analysis (PCA) of evolved and ancestral treatments, based on the Bray-Curtis index. While all samples are different from the ancestor, there are no systematic treatment specific differences in bacterial species composition. Colors denote the different treatments, with green for 22ºC evolved samples, blue 26ºC, red 32ºC and purple, samples from the fluctuating treatments.

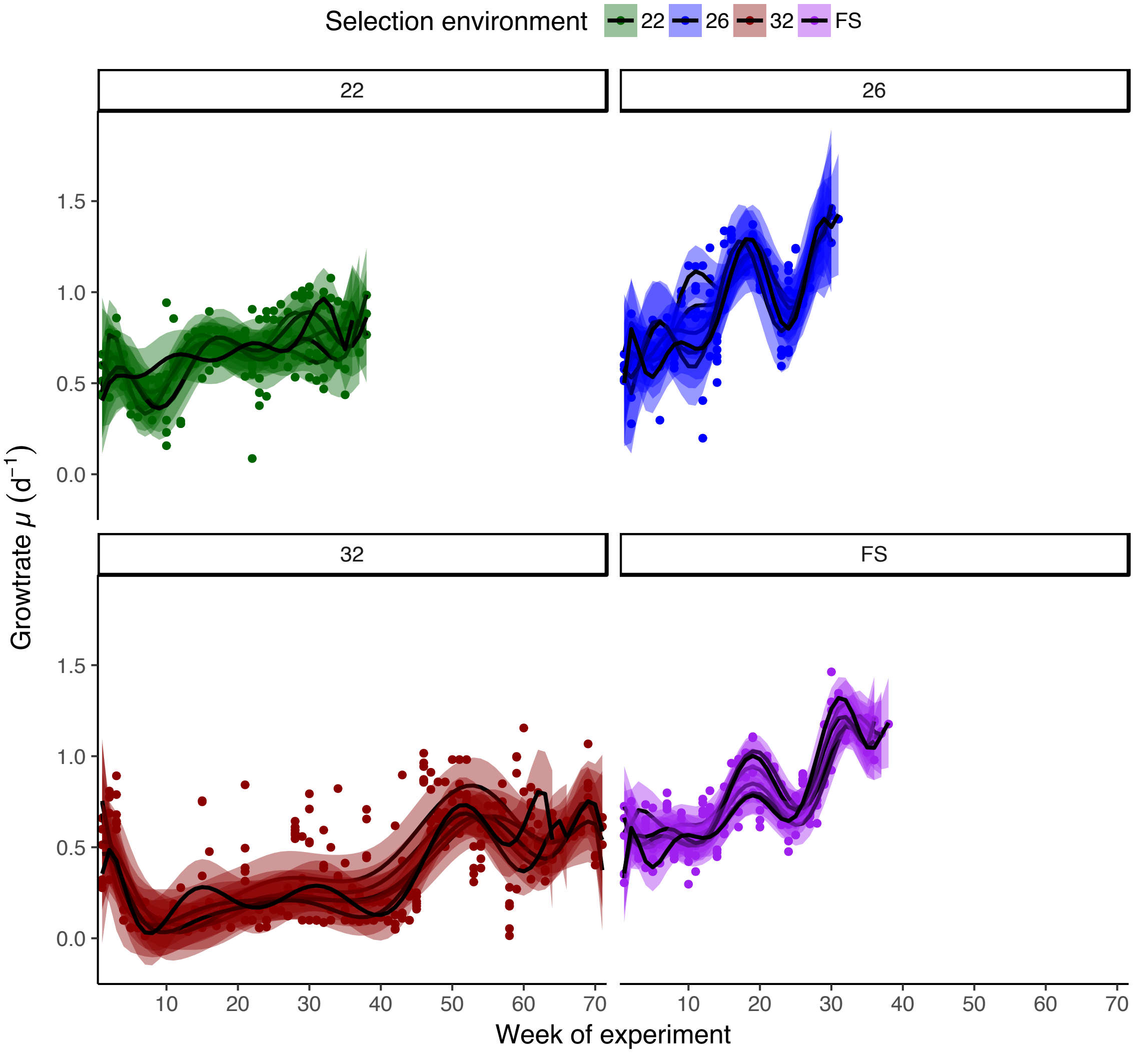

Supplementary Figure 15| Alternate version of Figure 1 (main manuscript) – per replicate trajectories of growth rate μ in the different treatments. While there is more variation between biological replicates in the 32ºC treatment than in any other treatment, the timing and magnitude of responses are conserved and repeatable within each treatment. Trajectories were fitted with a GAMM on a per-replicate level. Colors indicate the different selection regimes with green for 22ºC, blue for 26ºC, red for 32ºC and purple for the fluctuating treatment. Shaded areas around the GAMM are 1 s.e.

Supplementary Table 1| Summary of traits in ancestral and evolved populations. All ancestral trait values were measured at 22ºC. After 300 generations, they were measured in the evolved samples at the temperature of their selection environment. Responses for samples evolved in the fluctuating treatment were measured at 22ºC, 26ºC 32ºC. For acute responses of FS-evolved lineages (i.e. metabolic traits and their thermal responses), they are displayed for 32ºC to aid comparison with the populations experiencing constant severe warming. All data are reported as means ± 1 s.e.m. Abbreviations and acronyms are used as follows: C for carbon, N for nitrogen, P for phosphate, M, for the assimilation quotient of CO_2:O_2, *P* as gross photosynthesis (µgC µgC $^{-1}$d^{-1}), *R* as respiration (µgC µgC $^{-1}$ d^{-1}), NP as net photosynthesis (µgC µgC $^{-1}$ d^{-1}) taking into account 12 hours of photosynthesis and 24 of respiration, Φ_{PSII} as photosynthetic efficiency of PS II at 100µmol quanta m^{-2} s^{-1} (approximate light intensity in incubators), CUE as carbon use efficiency (1-*R*/*P* – as extracted from the thermal tolerance curves in Figure *2*) and the metabolic traits describing the shape of unimodal thermal reaction norms are E_a, $P(T_c)$, $R(T_c)$, T_h, E_h and T_{opt}.

		**Ancestor (at 22ºC)**	**22ºC**	**26ºC**	**32ºC**	**FS**
**Growth rate at t0 or t300**		0.63± 0.13	0.77 ± 0.06	1.36 ± 0.05	0.63 ± 0.05	1.1 ± 0.08
**Geometric mean growth rate**		0.63± 0.13	0.71 ± 0.08	1.08 ±0 .11	0.24 ± 0.11	0.87 ± 0.12
Cellular traits	**Chl:C** (mg:mg)	0.024 ±0.007	0.071 ± 0.002	0.121 ± 0.002	0.088 ± 0.007	0.085 ± 0.007
	**C:N** (mol:mol)	7.07 ± 0.07	6.94 ± 0.12	7.08 ± 0.08	7.21 ± 0.14	7.18 ± 0.21
	**C:P** (mol:mol)	70.78 ± 2.87	69.91 ± 2.75	84.91 ± 2.21	112.93 ± 3.71	93.22 ± 8.99
	**N:P** (mol:mol)	10.16 ± 0.33	10.14 ± 0.39	12.75 ± 0.41	15.07 ± 0.26	13.21 ± 0.55
	**M** (CN/CN+2)	0.779	0.778	0.780	0.783	0.782
	**C** (pmol/cell)	9.97± 1.09	13.87 ± 0.81	19.66 ± 0.82	18.37 ± 1.12	19.21 ± 0.75
	**N** (pmol/cell)	1.41 ± 0.14	1.98 ± 0.09	2.76 ± 0.13	2.53 ± 0.08	2.67±0.11
	**P** (pmol/cell)	0.139 ± 0.017	0.13± 0.009	0.216 ± 0.008	0.168 ± 0.019	0.202 ± 0.016
	**Silicate** (pmol /cell)	0.353 ± 0.061	0.317 ± 0.025	0.346 ± 0.023	0.168 ± 0.077	0.311 ± 0.02
	**Volume** (µm^3)	3794.25 ± 680.96	3610.65 ± 789.14	6359.75 ±818.17	13582.53 ± 692.95	4643.54 ± 673.61
Metabolic traits	***P***	19.95 ± 1.32	17.05 ± 3.25	10.14 ± 0.45	9.68 ± 0.11	9.95 ± 1.05
	R	9.97 ± 0.12	6.76 ± 1.59	2.01 ± 0.06	4.23 ± 1.61	2.67 ± 0.07
	NP	0.95 ± 0.04	1.86 ± 0.54	3.06± 0.27	0.61 ± 0.09	2.30 ± 0.60
	Φ_{PSII}	0.41 ± 0.005	0.27 ± 0.006	0.29 ± 0.01	0.13 ± 0.04	0.24 + 0.02
	**CUE**	0.54 ± 0.12	0.62 ± 0.04	0.81 ± 0.10	0.57 ± 0.03	0.74 ± 0.01
Thermal tolerance of growth	E_a (eV)	0.36 ± 0.19	0.25 ± 0.08	0.57 ± 0.1	0.57 ± 0.09	0.91 ± 0.1
	$\mu(T_c)$	-0.47 ± 0.11	-0.5 ± 0.11	-0.46 ± 0.15	-1.18 ± 0.16	-1.1 ± 0.16
	E_h(eV)	6.11 ± 2.95	4.8 ± 0.93	7.32 ± 1.29	2.86 ± 1.28	4.14 ± 1.29

	***T**_h* **(K)**	305.46 ± 1.77		No treatment effect 308.16 ± 0.35		
	**T_{opt} (ºC)**	28.69 ± 0.61	30.89 ± 0.16	32.14 ± 0.29	31.08 ± 0.62	33.90 ± 0.92
Thermal response of *P*	***E**_a* (eV)	1.07 ± 0.16		No treatment effect 0.87 ± 0.03		
	***P*(*T*_c)**	2.31 ± 0.12	2.29± 0.05	1.89 ± 0.09	2.10 ± 0.03	1.84 ± 0.09
	***E**_h*(eV)	3.51 ± 0.29	3.24 ± 0.28	3.17 ± 0.39	2.98 ± 0.36	2.13 ± 0.36
	***T**_h* (K)	303.41 ± 0.94	306.49 ± 0.55	306.49 ± 0.55	306.49 ± 0.55	306.49 ± 0.55
	**T_{opt}** (ºC)	27.27 ± 0.7	30.86 ± 0.02	32.03 ± 0.01	31.14 ± 0.04	33.37 ± 0.06
Thermal response of *R*	***E**_a* (eV)	1.07 ± 0.16		No treatment effect 0.83 ± 0.04		
	***R*(*T*_c)**	1.71 ± 0.09	1.41 ± 0.08	0.49 ± 0.03	1.12 ± 0.08	0.52 ± 0.02
	***E**_h* (eV)	2.54 ± 0.14	3.35 ± 0.46	2.66 ± 0.6	2.93 ± 0.52	1.74 ± 0.54
	***T**_h* (K)	305.25 ± 0.94		No treatment effect 307.74 ± 0.81		
	**T_{opt}** (ºC)	28.82± 0.36	31.99 ± 0.01	32.62± 62	31.83 ± 0.42	33.95 ± 0.92

Supplementary Table 2 | Model selection on generalised additive mixed effects model (GAMM) fitted to the trajectories of population growth. We fitted a GAMM to test whether the trajectories of population growth differed among the selection regimes. In the model, the effect of 'treatment' assesses whether median log-growth rates differ among selection regimes, while s(day.of.exp, by = selection regime) indicates whether the trajectories of growth rate differ among the selection regimes. Models were compared via the small sample-size corrected Akaike Information Criterion (AICc), delta AICc is the difference in AICc score relative to the model with the lowest value (most parsimonious model) and Weight is the relative support for the model. The best fitting models were selected as those returning the lowest AICc score and the highest AICc weight and are highlighted in bold.

Model selection table

formula = mue ~ treatment + s(day.of.exp, by = selection regime, bs='cr'), random = ~1 | selection regime/replicate

	**Intercept**	**s(day.of.exp, treatment)**	**selection regime**	**df**	**logLik**	**AICc**	**Delta**	**Weight**
**4 (full model)**	**0.71**	**+**	**+**	**15**	**276.1**	**-525.0**	**0**	**0.87**
2	0.73	+		12	271.99	-521.2	3.80	0.13
1	0.70			4	-50.26	399.0	923.93	0
3	0.66	+		7	-49.28	405.5	930.44	0

Supplementary Table 3 | Model selection on generalised additive mixed effects model (GAMM) fitted to the trajectories of population size. We fitted a GAMM to test whether the trajectories of population size differed among the selection regimes. In the model, the effect of 'treatment' assesses whether median log-population size differ among selection regimes, while s(day.of.exp, by = treatment) indicates whether the trajectories of growth population size differ among the selection regimes. Models were compared via the small sample-size corrected Akaike Information Criterion (AICc)), delta AICc is the difference in AICc score relative to the model with the lowest value (most parsimonious model) and Weight is the relative support for the model. The best fitting models were selected as those returning the lowest AICc score and the highest AICc weight and are highlighted in bold.

Model selection table

formula = pop size~ treatment + s(day.of.exp, by = selection regime, bs='cr'), random = ~ 1 | selection regime/replicate

	**Intercept**	**s(day.of.exp, treatment)**	**selection regime**	**df**	**Log Lik**	**AICc**	**Delta**	**Weight**
**4 (full model)**	7.018	+	+	**11**	-489.3	9809.5	0	0.927
2	7.16	+		8	-489.9	9814.6	5.07	0.073
3	6.905			7	-493.8	9891.4	81.88	0
1	6.998	+		4	-494.3	9894.4	84.92	0

Supplementary Table 4 | Thermal tolerance curve parameters for the ancestor. The thermal tolerance curve was quantified by fitting Eq. (6) to the growth rates quantified over a temperature gradient from 15ºC to 40ºC. CI (95%) are the lower and upper 95% confidence intervals.

Parameter	Environment	Estimate	CI (95%) [lower, upper]
$\mu(T_c)$	22	-0.47	[-1.12, -0.27]
E_a	22	0.35	[0.31, 1.13]
E_h	22	6.12	[6.04, 12.11]
T_h	22	305.46 K or 32.31ºC	[301.75, 309.1] or ºC [28.62, 36.01]

Supplementary Table 5 | Model selection and parameters of thermal tolerance curves of the evolved lineages. The mechanistic temperature dependence function (see Eq. (6), also Fig. 1) was fitted to the growth rate quantified over a temperature gradient from 15ºC to 40ºC for all evolved lineages (see Supplementary Table 4 for analysis of the ancestor). Models included random effects on each of the parameters of Eq. (6) by replicate and 'selection environment' as a fixed four level factor on each parameter. Models were compared via the small sample-size corrected Akaike Information Criterion (AICc), delta AICc is the difference in AICc score relative to the model with the lowest value (most parsimonious model) and Weight is the relative support for the model. The best fitting models were selected as those returning the lowest AICc score and the highest AICc weight and are highlighted in bold. In the model output, CI (95%) are the lower and upper 95% confidence intervals.

Model selection table

Model name	Remove selection environment effect on	K	AICc	Delta	Weight	Log lik
**resl.mix3**	$\boldsymbol{T_h}$	**18**	**218.81**	**0**	**0.78**	**-88.65**
resl.mix7	$E_h + T_h$	15	222.04	3.22	0.16	-94.13
resl.mix	NA - full model	21	223.81	4.99	0.06	-87.09
resl.mix11	$E_a + E_h + T_h$	12	239.43	20.62	0	-106.52
resl.mix6	Ea + T_h	15	239.93	21.11	0	-103.07
resl.mix4	$\mu(T_c)$	18	242.31	23.49	0	-100.39
resl.mix14	$\mu(T_c)+ E_h + T_h$	12	243.14	24.32	0	-108.37
resl.mix12	$\mu(T_c)+ E_a + E_h$	12	250.66	31.85	0	-112.13
resl.mix15	All	9	251.98	33.16	0	-116.31
resl.mix2	E_h	18	286.3	67.49	0	-122.39
resl.mix13	$\mu(T_c)+ E_a + T_h$	12	299.85	81.04	0	-136.73
resl.mix10	$\mu(T_c)+ T_h$	15	305.06	86.25	0	-135.64

resl.mix1 E_a - no convergence

resl.mix5 $E_a + E_h$ - no convergence

Model parameters

Parameter	Environment	Estimate	CI (95%) [lower, upper]
$\mu(T_c)$	22	-0.5	[-0.71,-0.29]
$\mu(T_c)$	26	-0.46	[-0.97,-0.01]
$\mu(T_c)$	32	-1.18	[-1.69,-0.66]
$\mu(T_c)$	FS	-1.1	[-1.62,-0.57]

E_a (eV)	22	0.25	[0.11,0.51]	
E_a(eV)	26	0.57	[0.21,0.91]	
E_a(eV)	32	0.57	[0.23, 0.91]	
E_a(eV)	FS	0.91	[0.57,1.25]	
E_h (eV)	22	4.8	[2.31,5.85]	
E_h (eV)	26	7.32	[3.28,12.13]	
E_h (eV)	32	2.86	[0.01,6.98]	
E_h (eV)	FS	4.14	[0.07,8.90]	
T_h	No treatment effect	308.16 K or 35.01ºC	[307.49,308.82]	or ºC [34.34, 35.67]

Supplementary Table 6 | Model selection and parameters for the thermal responses of gross photosynthesis and respiration in the ancestor. Eq. (6) was fitted to the metabolic rates quantified over a temperature gradient from 7ºC to 40ºC (3ºC increments) for the ancestor using a non-linear mixed effects model. "Flux", i.e. respiration or photosynthesis, was fitted as a fixed two-level factor to test for differences in thermal responses for photosynthesis (P) and respiration (R), and model selection otherwise proceeded as described above. Models were compared via the small sample-size corrected Akaike Information Criterion (AICc), delta AICc is the difference in AICc score relative to the model with the lowest value (most parsimonious model) and Weight is the relative support for the model. The best fitting models were selected as those returning the lowest AICc score and the highest AICc weight and are highlighted in bold.

Model selection

**Model name**	**Remove "flux" effect on**	**K**	**AICc**	**Delta**	**Weight**	**Log Lik**
Mod4	E_a	8	217.19	0	0.976	-100.595
Mod2	$P(T_c)$	9	225.44	8.25	0.015	-103.72
Mod1	E_h	10	227.62	10.43	0.005	-103.81
Mod3	T_h	8	229.01	11.82	0.002	-106.505

Parameters

**Parameter**	**Estimate**	**CI 95% [lower, upper]**	
E_a (eV)	1.07	[0.73,1.44]	
$P(T_c)$	2.31	[2.25,2.57]	
$R(T_c)$	1.71	[1.11,1.99]	
E_h. P (eV)	3.51	[3.17,4.39]	
E_h.R (eV)	2.54	[2.35,3.53]	
T_h. P (K)	303.41 (K) or 30.26ºC	[301.29,305.9]	or ºC [28.14,32.8]
T_h.R (K)	305.25 or 32.10ºC	[294.88,306.9]	or ºC [21.73,33.84]

Supplementary Table 7| Model selection and parameters for the thermal response of gross photosynthesis in the evolved lineages. Eq. (6) was fitted to the metabolic rates quantified over a temperature gradient from 7ºC to 40ºC (3ºC increments) for the evolved lineages using a non-linear mixed effects model. "Selection regime" was fitted as a fixed factor to test for differences in the parameters characterizing the thermal response for photosynthesis among the selection regimes. Models were compared via the small sample-size corrected Akaike Information Criterion (AICc), delta AICc is the difference in AICc score relative to the model with the lowest value (most parsimonious model) and Weight is the relative support for the model. The best fitting models were selected as those returning the lowest AICc score and the highest AICc weight and are highlighted in bold.

Model selection table

Model name	Remove selection regime effect on	K	AICc	Delta	Weight	Log Lik
**gp.mix6**	**$E_a + T_h$**	**14**	**300.28**	**0**	**0.46**	**-135.21**
gp.mix11	$E_a + E_h + T_h$	11	302.78	2.5	0.13	-139.81
gp.mix15	All	8	303.34	3.06	0.1	-143.36
gp.mix3	T_h	17	303.61	3.33	0.09	-133.43
gp.mix1	E_a	17	303.74	3.46	0.08	-133.5
gp.mix8	$P(T_c)+ E_a$	14	304.08	3.81	0.07	-137.11
gp.mix12	$P(T_c)+ E_a+ E_h$	11	305.4	5.12	0.04	-141.12
gp.mix7	$E_h+ T_h$	14	307.32	7.04	0.01	-138.73
gp.mix14	$P(T_c)+E_h + T_h$	11	307.72	7.44	0.01	-142.28
gp.mix	NA - full model	20	309.34	9.06	0	-132.76
gp.mix4	$P(T_c)$	17	309.64	9.36	0	-136.45
gp.mix2	E_h	17	310.05	9.78	0	-136.65
gp.mix13	$P(T_c)+E_a+T_h$	11	312.68	12.41	0	-144.77
gp.mix5	$E_a + E_h$ - no converfence					
gp.mix9	$P(T_c)+ E_h$ - no convergence					

Parameters

Treatment effect on	Environment	Estimate	CI (95%)
$P(T_c)$	22	2.29	[2.19,2.35]
$P(T_c)$	26	1.89	[1.70,2.16]
$P(T_c)$	32	2.10	[2.03,2.14]
$P(T_c)$	FS	1.84	[-1.74,1.92]
E_a (eV)	No treatment effect	0.87	[0.81,0.94]

E_h (eV)	22	3.24	[2.71, 3.82]	
E_h (eV)	26	3.17	[1.95, 4.55]	
E_h (eV)	32	2.98	[1.74,4.23]	
E_h(eV)	FS	2.13	[0.89, 3.41]	
T_h	No treatment effect	306.49 K (or 33.34ºC)	[303.42, 307.57]	Or ºC [30.27,34.42]

Supplementary Table 8 | Model selection and parameters for the thermal response of respiration in the evolved lineages. Eq. (6) was fitted to the metabolic rates quantified over a temperature gradient from 7ºC to 40ºC (3ºC increments) for the evolved lineages using a non-linear mixed effects model. "Selection regime" was fitted as a fixed factor to test for differences in the parameters characterizing the thermal response for respiration among the selection regimes. Models were compared via the small sample-size corrected Akaike Information Criterion (AICc), delta AICc is the difference in AICc score relative to the model with the lowest value (most parsimonious model) and Weight is the relative support for the model. The best fitting models were selected as those returning the lowest AICc score and the highest AICc weight and are highlighted in bold.

Model selection table

**Model name**	**Remove selection environment effect on**	**K**	**AICc**	**Delta**	**Weight**	**Log Lik**
**r.mix6**	$\boldsymbol{E_a + T_h}$	**15**	**279.94**	**0**	**0.64**	**-123.83**
**r.mix11**	$\boldsymbol{E_a + E_h + T_h}$	**12**	**281.84**	**1.9**	**0.25**	**-128.19**
r.mix1	E_a	18	284.95	5.01	0.05	-122.82
r.mix3	T_h	18	285.45	5.51	0.04	-123.07
r.mix7	$E_h + T_h$	15	288.51	8.57	0.01	-128.11
r.mix15	All	9	290.74	10.81	0	-135.96
r.mix	NA- full model	21	291.51	11.57	0	-122.49
r.mix8	$R(T_c)+E_a$	15	293.18	13.24	0	-130.45
r.mix12	$R(T_c)+ E_a + E_h$	12	293.77	13.83	0	-134.15
r.mix9	$R(T_c)+ E_h$	15	295.98	16.05	0	-131.85
r.mix14	$R(T_c)+ E_h + T_h$	12	297.2	17.26	0	-135.87
r.mix4	$R(T_c)$	18	299.26	19.32	0	-129.98
r.mix2	E_h	18	299.37	19.43	0	-130.03
r.mix10	$R(T_c)+T_h$	15	303.08	23.14	0	-135.4
r.mix13	$R(T_c)+ E_a + T_h$	12	306.02	26.08	0	-140.28
r.mix5	$E_a + E_h$ - no convergence					

Evolved samples - components of the two best models

	**df**	**logLik**	**AICc**	**Delta**	**weight**
r.mix6	15	-123.83	279.94	0	0.72
r.mix11	12	-128.19	281.84	1.9	0.28

Parameter estimates for Delta AICc <2

	$R(T_c)$	$\boldsymbol{E_a}$ **(eV)**	$\boldsymbol{E_h}$ **(eV)**	$\boldsymbol{T_h}$ **K**	$\boldsymbol{T_h}$ **ºC**

22 ºC	1.41	0.83 (no treatment effect)	3.35	307.47 (no treatment effect)	34.59 (no treatment effect)
26ºC	0.49		2.66		
32ºC	1.12		2.93		
FS	0.52		1.74		
	**Sum of AIC based relative weights**				
	$R(T_c)$	E_a **(eV)**	E_h **(eV)**	T_h	
	0.99	0.05	0.73	0.05	
	**95% interval**				
	$R(T_c)$	E_a **(eV)**	E_h **(eV)**	T_h K	T_h ºC
22 ºC	[1.25, 1.44]	[0.71,0.84]	[2.43,4.26]	[305.82,310.5]	[32.67,37.48]
26ºC	[0.34, 0.87]	(no treatment effect)	[0.54,4.78]	(no treatment effect)	
32ºC	[1.10, 1.23]		[0.96,4.87]		
FS	[0.31, 0.98]		[0.26,3.74]		

Supplementary Table 9 | Model selection to determine the effects of selection regime on the carbon use efficiency. We fitted the CUE data to a linear mixed model to test whether CUE differed among the selection regimes. Models were compared via the small sample-size corrected Akaike Information Criterion (AICc), delta AICc is the difference in AICc score relative to the model with the lowest value (most parsimonious model) and Weight is the relative support for the model. The best fitting models were selected as those returning the lowest AICc score and the highest AICc weight and are highlighted in bold. The best fitting model included differences in CUE among the selection regimes.

**Model selection table**							
Formula	fixed = cue ~ selection regime, random = ~1 | selection regime/replicate						
**Model**	**Intercept**	**selection regime**	**Df**	**logLik**	**AICc**	**Delta**	**weight**
**2**	**0.63**	+	**7**	**35.84**	**-53.70**	**0.00**	**1.00**
1	0.68		3	22.37	-38.00	15.69	0.00

Parameter estimates and 95% Confidence intervals

**Selection regime**	**Parameter Estimate**	**95% Confidence interval [lower, upper]**
Ancestor	0.63	[0.59, 0.67]
22ºC	0.71	[0.65, 0.77]
26ºC	0.81	[0.77, 0.85]
32ºC	0.67	[0.61, 0.73]
FS	0.71	[0.69, 0.73]

Supplementary Table 10| Model selection for size, C, N, P, Si, RNA, and protein quota per cell volume as well as C:N, C:P, N:P, Chl:C ratio, and Φ_{PSII} at irradiance as in the incubator for ancestral samples. All traits were analyzed using separate mixed effects models, where 'assay temperature' ranging from 15ºC to 35ºC was a fixed effect and replicate nested within temperature was a random effect. In all traits, there was a significant effect of the assay temperature on the tait value. Model selection was carried out based on lowest AICc score and are highlighted in bold. Parameter estimates and 95% confidence intervals are presented below.

Model selections

Cell volume (μm^3)

Global Model: fixed = Size ~ assay temp, random = ~1 | replicate

Model	Chisq	Assay T	Df	logLik	AICc	delta	p
**1**	**59.13**	**+**	**9**	**-14.14**	**46.29**	**0**	**<0.001**
2			3	-43.71	93.42	47.13	

C (pmol) per cell volume

: fixed = Cpervolume ~ assay temp, random = ~1 | replicate

Model	Chisq	Assay T	Df	logLik	AICc	delta	p
**1**	**55.76**	**+**	**9**	**306.21**	**-594.42**	**0**	**<0.001**
2			3	278.33	-550.66	43.76	

N (pmol) per cell volume

Global Model: fixed = Npervolume~ assay temp, random = ~1 | replicate

Model	Chisq	Assay T	Df	logLik	AICc	delta	p
**1**	**43.78**	**+**	**9**	**381.66**	**-745.31**	**0**	**<0.001**
2			3	359.76	-713.53	31.73	

P (pmol) per cell volume

Global Model: fixed = Ppervolume~ assay temp, random = ~1 | replicate

Model	Chisq	Assay T	Df	logLik	AICc	delta	p
**1**	**37.44**	**+**	**9**	**508.74**	**-999.48**	**0**	**<0.001**
2			3	490.02	-974.03	25.45	

Total protein (ng) per cell volume

Global Model: fixed = Protpervolume~ assay temp, random = ~1 | replicate

Model	Chisq	Assay T	Df	logLik	AICc	delta	p
**1**	**46.41**	**+**	**9**	**578.35**	**-1138.7**	**0**	**<0.001**
2			3	555.15	-1104.3	34.4	

Total RNA(pg) per cell volume

Global Model: fixed = RNApervolume~ assay temp, random = ~1 | replicate							
Model	Chisq	Assay T	Df	logLik	AICc	delta	p
**1**	**75.07**	+	**9**	**308.32**	**-598.65**	**0**	**<0.001**
2			3	270.79	530.51	63.08	

Silicate (pmol) per cell volume

Global Model: fixed = Si ~assay tempcate							
Model	Chi	AICc	delta	p			
**1**	**22.74**	+	**9**	**306.39**	**-594.79**	**0**	**<0.001**
2			3	295.02	-584.04	10.75	

C:N

Global Model: fixed = CN ~assay temp, random = ~1| replicate							
Model	Chisq	Assay T	Df	logLik	AICc	delta	p
**1**	**17.12**	+	**9**	**-44.78**	**107.6**	**0**	**<0.01**
2			3	-53.65	113.30	5.73	

N:P

Global Model: fixed = NP ~assay temp, random = ~1| replicate							
Model	Chisq	Assay T	Df	logLik	AICc	delta	p
**1**	**33.21**	+	**9**	**-96.55**	**211.11**	**0**	**<0.001**
2			3	-113.11	232.32	22.11	

C:P

Global Model: fixed = CN ~assay temp, random = ~1| replicate							
Model	Chisq	Assay T	Df	logLik	AICc	delta	p
**1**	**60.12**	+	**9**	**-163.18**	**344.62**	**0**	**<0.001**
2			6	-193.24	392.48	48.12	

Chlorophyll:C ratio

Global Model: fixed = Chl:C ~assay temp, random = ~1 | selection regime/replicate							
Model	Chisq	Assay T	Df	logLik	AICc	delta	p
**1**	**50.12**	+	**9**	**413.58**	**-121.84**	**0**	**<0.001**
2			6	352.88	-83.72	38.3	

Φ_{PSII}

Global Model: fixed = Φ_{PSII} ~ assay temp, random = ~1 | replicate							
Model	Chisq	Assay T	Df	logLik	AICc	delta	p
**1**	**13.71**	+	**4**	**16.44**	**-24.81**	**0**	**<0.01**

2	3	9.15	-13.10	11.71

Parameter estimates and lower and upper confidence intervals ('conf')

Size in µm

Assay at	Estimate	conf lower	conf upper
15ºC	11.54	11.26	11.83
20ºC	22.13	11.26	0.00
22ºC	21.99	11.26	0.00
25ºC	21.99	11.26	0.00
30ºC	21.52	11.26	0.00
32ºC	21.19	11.26	0.00
35ºC	20.99	11.26	0.00

C (pmol) per cell volume

Assay at	Estimate	conf lower	conf upper
15ºC	1.79E-04	8.54E-05	2.73E-04
20ºC	2.32E-04	5.51E-06	4.59E-04
22ºC	2.27E-04	5.43E-08	4.54E-04
25ºC	2.71E-04	4.42E-05	4.98E-04
30ºC	5.55E-04	3.28E-04	7.81E-04
32ºC	6.04E-04	3.77E-04	8.31E-04
35ºC	7.42E-04	5.00E-04	9.84E-04

N (pmol) per cell volume

Assay at	Estimate	conf lower	conf upper
15ºC	3.01E-05	1.59E-05	4.43E-05
20ºC	3.04E-05	-3.97E-06	6.48E-05
22ºC	3.37E-05	-6.86E-07	6.81E-05
25ºC	3.51E-05	6.71E-07	6.95E-05
30ºC	7.77E-05	4.33E-05	1.12E-04
32ºC	7.53E-05	4.09E-05	1.10E-04
35ºC	9.50E-05	5.82E-05	1.32E-04

P (pmol) per cell volume

Assay at	Estimate	conf lower	conf upper
15ºC	2.8E-06	1.6E-07	1.8E-06
20ºC	3.8E-06	3.3E-07	3.9E-06
22ºC	4.0E-06	5.2E-07	3.6E-06

25ºC	3.8E-06	2.6E-07	4.8E-06
30ºC	5.0E-06	1.5E-06	5.0E-06
32ºC	5.1E-06	1.6E-06	5.8E-06
35ºC	5.8E-06	2.2E-06	1.8E-06

Protein (ng) per cell volume

Assay at	Estimate	conf lower	conf upper
15ºC	1.67E-07	6.26E-08	2.71E-07
20ºC	3.38E-07	8.57E-08	2.16E-06
22ºC	3.35E-07	8.33E-08	2.16E-06
25ºC	4.89E-07	2.37E-07	2.31E-06
30ºC	7.14E-07	4.62E-07	2.54E-06
32ºC	6.92E-07	4.40E-07	2.52E-06
35ºC	4.93E-07	2.24E-07	2.34E-06

RNA (pg) per cell volume

Assay at	Estimate	conf lower	conf upper
15ºC	2.82E-04	1.93E-04	3.71E-04
20ºC	3.45E-04	1.30E-04	5.60E-04
22ºC	2.82E-04	6.72E-05	4.97E-04
25ºC	2.88E-04	7.27E-05	5.03E-04
30ºC	6.02E-04	3.87E-04	8.18E-04
32ºC	7.49E-04	5.33E-04	9.64E-04
35ºC	1.05E-03	8.19E-04	1.28E-03

Silicate (pmol) per cell volume

Assay at	Estimate	conf lower	conf upper
15ºC	1.71E-04	7.71E-05	2.65E-04
20ºC	3.12E-04	9.10E-05	5.36E-04
22ºC	1.02E-04	-1.19E-04	3.23E-04
25ºC	1.90E-04	-3.10E-05	4.11E-04
30ºC	4.88E-05	-1.72E-04	2.70E-04
32ºC	2.37E-05	-1.97E-04	2.45E-04
35ºC	1.17E-05	-2.20E-04	2.51E-04

C:N

Assay at	Estimate	conf lower	conf upper
15ºC	6.42	5.80	7.03
20ºC	7.61	6.21	9.06
22ºC	6.80	5.40	8.21

25ºC	7.76	6.36	9.17
30ºC	7.33	5.93	8.74
32ºC	7.99	6.59	9.40
35ºC	7.78	6.36	9.36

N:P

Assay at	Estimate	conf lower	conf upper
15ºC	10.87	8.66	13.09
20ºC	8.11	7.76	11.13
22ºC	8.37	7.02	12.73
25ºC	9.37	7.66	14.73
30ºC	15.27	9.91	16.72
32ºC	14.69	9.33	18.04
35ºC	15.86	10.13	20.02

Chl:C

Assay at	Estimate	conf lower	conf upper
15ºC	5.77E-02	2.31E-02	9.23E-02
20ºC	9.68E-02	1.39E-02	1.80E-01
22ºC	2.05E-01	1.23E-01	2.88E-01
25ºC	1.85E-01	1.02E-01	2.68E-01
30ºC	1.11E-01	2.77E-02	1.93E-01
32ºC	3.44E-02	1.00E-03	1.17E-01
35ºC	1.22E-02	1.00E-04	1.00E-01

ΦPSII

Assay at	Estimate	conf lower	conf upper
15ºC	NA	NA	NA
20ºC	NA	NA	NA
22ºC	0.38	0.32	0.4
25ºC	0.39	0.32	0.42
30ºC	NA	NA	NA
32ºC	0.093	0.085	0.1
35ºC	0.088	0.024	0.099

C:P

Assay at	Estimate	conf lower	conf upper
15ºC	61.88	50.72	74.65
20ºC	56.78	34.09	89.67
22ºC	72.44	68.98	84.57
25ºC	110.44	84.71	98.20

30ºC	117.72	84.99	136.01
32ºC	122.63	92.27	143.49
35ºC	62.68	97.69	111.84

Supplementary Table 11| Model selection for size, C, N, P, Si, RNA, and protein quota per cell volume as well as C:N, C:P, N:P, Chl:C ratio, and Φ_{PSII} at irradiance as in the incubator for samples after 300 generations of selection. All traits were analyzed using separate mixed effects models, where 'selection regime' was a fixed effect and replicate nested within selection regime was a random effect on the intercept. In all traits, there was a significant effect of selection regime. Models were compared via the small sample-size corrected Akaike Information Criterion (AICc), delta AICc is the difference in AICc score relative to the model with the lowest value (most parsimonious model) and Weight is the relative support for the model. The best fitting models were selected as those returning the lowest AICc score and the highest AICc weight and are highlighted in bold.

Model selection

Cell volume (µm^3)

Global Model: fixed = Size ~ selection regime, random = ~1 | selection regime/replicate

Model	Intercept	selection regime	Df	logLik	AICc	delta	weight
**2**	**3794**	+	**7**	**-2032.12**	**4078.8**	**0**	**1**
1	5776		3	-2048.43	4103	24.17	0

Carbon (pmol) per cell volume

Global Model: fixed = Ccellvolume ~ selection regime, random = ~1 | selection regime/replicate

Model	Intercept	selection regime	Df	logLik	AICc	delta	weight
**2**	**2.83E-04**	+	**7**	**208.70**	**-396.40**	**0.00**	**1**
1	2.27E-04	4	4	191.55	-373.00	23.39	0

Nitrogen (pmol) per cell volume

Global Model: fixed = Ncellvolume~ selection regime, random = ~1 | selection regime/replicate

Model	Intercept	selection regime	Df	logLik	AICc	delta	weight
**2**	**4.37E-05**	+	**7**	**250.285**	**-479.6**	**0**	**1**
1	3.71E-05		4	235.83	-461.6	18.02	0

Phosphorus (pmol) per cell volume

Global Model: fixed = Pcellvolume ~ selection regime, random = ~1 | selection regime/replicate

Model	Intercept	selection regime	Df	logLik	AICc	delta	weight
**2**	**4.66E-06**	+	**7**	**306.35**	**-591.7**	**0**	**1**
1	3.35E-06	4	4	289.702	-569.3	22.4	0

**Protein (ng) content per cell volume**							
Global Model: fixed = Protcellvolume ~ selection regime, random = ~1 | selection regime/replicate							
Model	Intercept	selection regime	Df	logLik	AICc	delta	weight
**2**	**3.35E-07**	+	**7**	**360.069**	**-699.1**	**0**	**0.842**
1	3.57E-07		4	352.946	-695.8	3.35	0.158

**RNA (pg) content per cell volume**							
Global Model: fixed = RNAcellvolume~ selection regime, random = ~1 | selection regime/replicate							
Model	Intercept	selection regime	Df	logLik	AICc	delta	weight
**2**	**2.62E-04**	+	**7**	**208.45**	**-395.9**	**0**	**0.998**
1	1.76E-04		4	196.834	-383.6	12.34	0.002

**Silicate (pmol) per cell volume**							
Global Model: fixed = Si ~ selection regime, random = ~1 | selection regime/replicate							
Model	Intercept	selection regime	Df	logLik	AICc	delta	weight
**2**	**5.88E-05**	+	**7**	**212.688**	**-411.1**	**0**	**0.51**
1	3.90E-05		4	216.045	-415.3	4.18	0.49

**C:N**							
Global Model: fixed = CN ~ selection regime, random = ~1 | selection regime/replicate							
Model	Intercept	selection regime	Df	logLik	AICc	delta	weight
**2**	**7.07**	+	**7**	**-76.05**	**167.50**	**0**	**0.99**
1	7.18		3	-88.973	183.60	16.07	0.01

**N:P**							
Global Model: fixed = NP ~ selection regime, random = ~1 | selection regime/replicate							
Model	Intercept	selection regime	Df	logLik	AICc	delta	weight
**2**	**10.16**	+	**7**	**-195.04**	**405.40**	**0**	**1.00**
1	12.46		3	-209.91	426.1	20.66	0

**C:P**							
Global Model: fixed = CP ~ selection regime, random = ~1 | selection regime/replicate							
Model	Intercept	selection regime	Df	logLik	AICc	Delta	weight
**2**	**70.77**	+	**7**	**-393.15**	**801.70**	**0.00**	**1.00**
1	90.72		3	-406.84	820	18.27	0.00

**Chlorophyll:C ratio**							
Global Model: fixed = Chl:C ~ selection regime, random = ~1 | selection regime/replicate							
Model	Intercept	selection regime	Df	logLik	AICc	delta	weight
**2**	**0.02**	**+**	**7**	**413.58**	**-812.6**	**0**	**1**
1	0.08		3	352.88	-699.6	112.95	0

**Φ_{PSII}**							
Global Model: fixed = Φ_{PSII} ~ selection regime, random = ~1 | selection regime/replicate							
Model	Intercept	selection regime	Df	logLik	AICc	delta	weight
**2**	**0.12**	**+**	**6**	**137.86**	**-263.71**	**0**	**0.81**
1	0.15		3	124.28	-242.56	21.15	0.19

Parameter estimates and 95% confidence intervals

**Cell volume (µm^3)**		
**Selection regime**	**Parameter estimate**	**95% Confidence interval [lower, upper]**
Ancestor	3794.25	[3113.64, 4474.86]
22ºC	3610.65	[2821.51, 4399.79]
26ºC	6359.75	[5541.58, 7177.92]
32ºC	8588.82	[7895.87, 9281.77]
FS	4643.54	[3969.93, 5317.15]

**C (pmol) per cell volume**		
**Selection regime**	**Parameter estimate**	**95% Confidence interval [lower, upper]**
Ancestor	2.27E-04	[1.22E-04, 3.32E-04]
22ºC	2.83E-04	[2.39E-04, 3.26E-04]
26ºC	2.67E-04	[1.62E-04, 3.72E-04]
32ºC	1.04E-04	[-3.23E-07, 2.09E-04]
FS	2.52E-04	[1.47E-04, 3.57E-04]

**N (pmol) per cell volume**		
**Selection regime**	**Parameter estimate**	**95% Confidence interval [lower, upper]**
Ancestor	-9.99E-06	[1.59E-05, 5.15E-05]
22ºC	4.37E-05	[3.63E-05, 5.11E-05]
26ºC	8.65E-07	[2.67E-05, 6.24E-05]
32ºC	-2.54E-05	[4.81E-07, 3.61E-05]

FS	-1.82E-06	[2.41E-05, 5.97E-05]

P (pmol) per cell volume

Selection regime	Parameter estimate	95% Confidence interval [lower, upper]
Ancestor	-6.35E-07	[2.54E-06, 5.50E-06]
22ºC	4.66E-06	[4.03E-06, 5.29E-06]
26ºC	-1.05E-06	[2.13E-06, 5.09E-06]
32ºC	-3.24E-06	[-5.98E-08, 2.90E-06]
FS	-9.63E-07	[2.22E-06, 5.18E-06]

Protein (ng) per cell volume

Selection regime	Parameter estimate	95% Confidence interval [lower, upper]
Ancestor	1.53E-10	[1.84E-07, 4.86E-07]
22ºC	3.35E-07	[2.69E-07, 4.01E-07]
26ºC	-1.50E-08	[1.69E-07, 4.71E-07]
32ºC	-3.06E-08	[1.53E-07, 4.55E-07]
FS	1.33E-07	[3.17E-07, 6.19E-07]

RNA (pg) per cell volume

Selection regime	Parameter estimate	95% Confidence interval [lower, upper]
Ancestor	2.08E-05	[1.93E-04, 3.72E-04]
22ºC	2.62E-04	[2.24E-04, 2.99E-04]
26ºC	-1.15E-04	[5.73E-05, 2.37E-04]
32ºC	-9.10E-05	[8.09E-05, 2.60E-04]
FS	-1.38E-04	[3.40E-05, 2.13E-04]

Silicate (pmol) per cell volume

Selection regime	Parameter estimate	95% Confidence interval [lower, upper]
Ancestor	4.33E-05	[3.96E-05, 1.65E-04]
22ºC	5.88E-05	[3.19E-05, 8.56E-05]
26ºC	-3.45E-05	[-3.82E-05, 8.68E-05]
32ºC	-3.81E-05	[-4.18E-05, 8.31E-05]
FS	-6.57E-06	[-1.03E-05, 1.15E-04]

C:N

Selection regime	Parameter estimate	95% Confidence interval [lower, upper]
Ancestor	7.07	[7.02, 7.12]
22ºC	6.94	[6.82, 7.06]

26ºC	7.08	[7.07, 7.09]
32ºC	7.21	[7.08, 7.34]
FS	7.18	[7.09, 7.27]

N:P

Selection regime	Parameter estimate	95% Confidence interval [lower, upper]
Ancestor	10.16	[9.93, 10.39]
22ºC	10.14	[9.85, 10.43]
26ºC	12.75	[12.44, 13.06]
32ºC	15.07	[14.81, 15.33]
FS	13.21	[12.76, 13.66]

C:P

Selection regime	Parameter estimate	95% Confidence interval [lower, upper]
Ancestor	70.78	[67.91, 73.65]
22ºC	69.91	[67.16, 72.66]
26ºC	84.91	[82.25, 87.57]
32ºC	112.93	[109.22, 116.64]
FS	93.22	[84.32, 102.12]
FS	0.202	[0.186, 0.218]

Chlorophyll:C ratio

Selection regime	Parameter estimate	95% Confidence interval [lower, upper]
Ancestor	0.024	[0.017, 0.031]
22ºC	0.071	[0.069, 0.073]
26ºC	0.121	[0.119, 0.123]
32ºC	0.088	[0.082, 0.094]
FS	0.085	[0.078, 0.092]

Φ_{PSII} at Iopt

Selection regime	Parameter estimate	95% Confidence interval [lower, upper]
Ancestor	0.12	[0.119, 0.121]
22ºC	0.09	[0.082, 0.098]
26ºC	0.12	[0.112, 0.128]
32ºC	0.08	[0.073, 0.087]
FS	0.12	[0.119, 0.121]

Supplementary Table 12| PERMANOVA and pairwise comparisons based on treatment-level divergence in SNVs. Using only single nucleotide variations(SNVs) that had reached fixation after 300 generations of evolution in the respective environment, distance matrix was calculated from Euclidean distances and passed to permutational multivariate analysis of variance (PERMANOVA) to assess overall treatment effects and individual pairwise differences between levels of the treatment were assessed with TukeyHSD tests, where CI denotes the confidence intervals, and , the associated p values.

**ANOVA Table**	**Phenotype**					
Response:	Distances					
	Df	Sum Sq	Mean Sq	F value	Pr(>F)	
Treatment	4	8.57	2.15	3.81	0.01845	*
Residuals	20	11.25	0.56			

**Pairwise distances**				
Comparison	Difference	Lower 95% CI	Upper 95% CI	P adj
26-22	0.049	0.037	0.056	0.041
32-22	0.023	0.014	0.055	0.051
Anc-22	-0.144	-0.236	-0.080	0.034
FS-22	0.097	0.081	0.102	0.047
32-26	-0.081	-0.091	-0.05	0.038
Anc-26	-0.073	-0.083	-0.019	0.033
FS-26	-0.031	-0.171	0.054	0.827
Anc-32	-0.148	-0.204	-0.110	0.042
FS-32	0.01	-0.01	0.018	0.050
FS-Anc	0.064	0.052	0.075	0.020

Supplementary Table 13| PERMANOVA and pairwise comparisons based on treatment-level divergence in phenotypic traits. Phenotypic trait values of a population in its selection environment after 300 generations of evolution were normalised relative to the trait values of the ancestor in that same environment. The traits investigated were gross photosynthesis at saturating light intensity and incubator light intensity, growth and respiration rates, intracellular stoichiometry (ratios and amounts per cell), cell size, chlorophyll content, FRRF data (dark adapted F_v/F_m at incubator and saturating light intensity, and as a function of light intensity for photosynthetic efficiency, relative rate of electron transport through PSII, *C* as the proportion of PSII reaction centres in a closed state, and NPQ as non-photochemical quenching), and flow cytometry data (side scatter for granularity, FL1 fluorescence after a rhodamine dye as a proxy for H+ transport across mitochondrial membranes, FL2 and FL3 fluorescence after a Nile Red dye as a proxy for intracellular lipid content).The phenotypic trait data were then analysed through calculating a difference matrix and using permutational multivariate analysis of variance (PERMANOVA) to assess overall treatment effects and individual pairwise differences between levels of the treatment were assessed with TukeyHSD tests.

**ANOVA Table**	**Phenotype**					
Response:	Distances					
	Df	Sum Sq	Mean Sq	F value	Pr(>F)	
Treatment	4	3.3427	0.83568	3.3174	0.03075	*
Residuals	20	5.0382	0.25191			

**Pairwise distances**				
Comparison	Difference	Lower 95% CI	Upper 95% CI	P
26-22	0.69	0.17	1.56	0.051
32-22	0.38	0.19	1.25	0.049
Anc-22	-1.04	-2.66	-0.28	0.034
FS-22	0.39	0.18	1.25	0.047
32-26	-0.31	-1.18	-0.15	0.048
Anc-26	-1.73	-3.35	-0.11	0.033
FS-26	-0.31	-1.17	0.56	0.827
Anc-32	-1.42	-3.04	-0.20	0.010
FS-32	0.01	-0.86	0.88	0.100
FS-Anc	1.43	0.82	3.05	0.020

Supplementary Table 14| Table of candidate genes in the populations from the fluctuating environment where variants reoccurred in independent replicate cultures subjected to elevated temperature but not those grown at moderate temperature. Listed below are candidate genes, the populations they occurred in, and putative function as retrieved through GO terms for biological processes where known. Note that this list is too small to carry out enrichment tests with confidence.

Gene	Population	Putative function
THAPSDRAFT_20620	FS b1 and FS b5	hypothetical protein, involved in transcription
THAPSDRAFT_23072	FS b1 and FS b5	hypothetical protein, protein coding
THAPSDRAFT_2335	FS b1 and FS b5	hypothetical protein, involved in transcription
THAPSDRAFT_24141	FS b1 and FS b5	hypothetical protein, involved in transcription
THAPSDRAFT_1762	FS b3 and FS b5	hypothetical protein, membrane traffic
THAPSDRAFT_21967	FS b1 and FS b3	hypothetical protein, zinc finger involved in transcription
THAPSDRAFT_2720	FS b1 and FS b3	hypothetical protein, protein coding, especially heat stress transcription

Supplementary Table 15| Aligned sequence depths for each sequenced population. After trimming and filtering, remaining sequence reads were then aligned against version 2 of the reference *T. pseudonana* genome sequence (GenBank: GCA_000149405.2) using BWA-mem version 0.7.5a-2 with default settings . This resulted in average aligned sequence depths of 18.5 X. and a set of 64 BAM-formatted files. Depths and insert lengths were calculated using Qualimap.

Population name	Mean aligned sequence depth (X)	Accession number	Mapping quality mean	Median insert length (b.p.)
t0_S45	18.46	All data can be found at SRA: SRP114919 BioProject: PRJNA397360	57.47	317.0
t300_22_b1_S19	18.39		57.54	281.0
t300_22_b2_S20	18.61		57.48	339.0
t300_22_b3_S21	17.66		57.6	259.0
t300_22_b4_S22	17.57		57.61	229.0
t300_22_b5_S23	18.52		57.45	333.0
t300_22_b6_S24	18.29		57.58	274.0
t300_26_b1_S25	18.65		52.83	345.0
t300_26_b2_S26	6.67		57.63	170.0
t300_26_b3_S27	15.02		57.72	109.0
t300_26_b4_S28	15.25		57.59	120.0
t300_26_b5_S29	18.71		57.48	353.0
t300_26_b6_S14	18.61		57.38	439.0
t300_32_b1_S3	18.65		57.53	431.0
t300_32_b2_S4	18.75		57.35	502.0
t300_32_b3_S5	18.68		57.45	489.0
t300_32_b4_S10	18.58		57.38	441.0
t300_32_b5_S9	18.41		57.4	391.0
t300_32_b6_S8	18.28		57.35	348.0
t300_FS_b1_S31	8.71		57.51	276.0
t300_FS_b2_S7	18.62		57.39	426.0
t300_FS_b3_S33	7.11		57.42	307.0
t300_FS_b4_S6	18.74		57.36	509.0
t300_FS_b5_S35	15.83		57.45	319.0
t300_FS_b6_S36	18.62		57.5	290.0

Supplementary Table 16| Model selection for the light response curves of photochemical efficiency. An exponential decay function (see Eq. (8)) was fitted to the photochemical efficiency (ϕ_{PSII}) light response curves using a non-linear mixed effects model. "Selection regime" was fitted as a fixed factor to test for differences in the parameters characterizing the light response curves for between the ancestor and the selection regimes. Models were compared via the small sample-size corrected Akaike Information Criterion (AICc), delta AICc is the difference in AICc score relative to the model with the lowest value (most parsimonious model) and Weight is the relative support for the model. The best fitting models were selected as those returning the lowest AICc score and the highest AICc weight and are highlighted in bold.

Model selection for ϕ_{PSII} ancestor populations

Model	Df	Assay temperature effect dropped on	AICc	Log Lik	Delta	Weight
**Full**	**9**		**-1087.66**	**552.83**	**0**	**0.74**
Exp.mix2	7	Slope b	-956.38	485.19	131.28	0.24
Exp.mix1	7	Intercept a	-695.53	354.76	392.21	0.02

Model selection for ϕ_{PSII} evolved populations

Model	Df	Selection regime effect dropped on	AICc	LogLik	Delta	Weight
**Full**	**11**		**-1554.52**	**-1512.54**	**0**	**0.84**
Exp.mix1	8	Intercept a	-1540.76	-1510.21	13.76	0.09
Exp.mix2	8	Slope b	-1540.39	-1509.85	14.13	0.07

Parameter estimates and 95% confidence intervals

**Selection regime and parameter**	**Estimate**	**95% confidence interval [lower, upper]**
Slope Ancestor (at 22ºC)	- 0.0013	[-0.0014, -0.0012]
Intercept Ancestor (at 22º)	0.51	[0.49, 0.53]
Slope Ancestor (at 26ºC)	- 0.0009	[-0.001, -0.0008]
Intercept Ancestor (at 26ºC)	0.31	[0.29, 0.32]
Slope Ancestor (at 32ºC)	- 0.0029	[-0.005, -0.0009]
Intercept Ancestor (at 32ºC)	0.21	[0.19, 0.23]
Slope evolved 22ºC	-0.0012	[-0.0014, -0.0010]
Intercept evolved 22ºC	0.36	[0.32, 0.40]

Slope evolved 26ºC	-0.0009	[-0.0011, -0.0007]
Intercept evolved 26ºC	0.41	[0.39, 0.43]
Slope evolved 32ºC	-0.0005	[-0.0006, -0.0004]
Intercept evolved 32ºC	0.56	[0.54, 0.58]
Slope evolved FS	-0.0008	[-0.0007, -0.0009]
Intercept evolved FS	0.46	[0.44, 0.48]

Supplementary Table 17| PERMANOVA and pairwise comparison for differences between treatments (Bacteria)

To estimate the relative abundances of taxa represented in the data, we used BLASTN (version 2.5.0+) to align 10 000 sequence reads from each sample against the NCBI's non-redundant Nucleotide database and assigned matches to species using MEGAN (version 5.11.3). A distance matrix was then calculated from Bray-Curtis distances and passed to permutational multivariate analysis of variance (PERMANOVA) to assess overall treatment effects and individual pairwise differences between levels of the treatment were assessed with TukeyHSD tests.

**ANOVA Table**	**SNPs**				
Response:	Distances				
	Df	Sum Sq	Mean Sq	F value	Pr(>F)
Treatment	4	0.0097	0.002	0.62	0.11
Residuals	20	0.079	0.004		

**Pairwise distances**				
Comparison	Difference	Lower 95% CI	Upper 95% CI	P
26-22	-0.028	-0.137	0.080	0.930
32-22	-0.022	-0.131	0.086	0.970
Anc-22	-0.181	-0.268	0.037	0.048
FS-22	0.013	-0.095	0.121	0.996
32-26	0.006	-0.102	0.114	1.000
Anc-26	-0.068	-0.239	0.006	0.038
FS-26	0.0415	-0.067	0.150	0.780
Anc-32	-0.098	-0.245	0.060	0.038
FS-32	0.036	-0.073	0.144	0.862
FS-Anc	-0.199	-0.124	0.002	0.046
